# Low-Level Antimicrobials in the Medicinal Leech Select for Resistant Pathogens That Spread to Patients

**DOI:** 10.1128/mBio.01328-18

**Published:** 2018-07-24

**Authors:** Lidia Beka, Matthew S. Fullmer, Sophie M. Colston, Michael C. Nelson, Emilie Talagrand-Reboul, Paul Walker, Bradley Ford, Iain S. Whitaker, Brigitte Lamy, Johann Peter Gogarten, Joerg Graf

**Affiliations:** a Department of Molecular and Cell Biology, University of Connecticut, Storrs, Connecticut, USA; b Équipe Pathogènes Hydriques Santé Environnements, UMR 5569 HSM, Université de Montpellier, Montpellier, France; c Département d’Hygiène Hospitalière, CHRU de Montpellier, Montpellier, France; d Department of Pathology, University of Iowa Hospitals and Clinics, Iowa City, Iowa, USA; e Institute of Life Sciences, Swansea University College of Medicine, Swansea, Wales, United Kingdom; f Laboratoire de Bactériologie, CHRU de Montpellier, Montpellier, France; g Institute for Systems Genomics, University of Connecticut, Storrs, Connecticut, USA; h INSERM U1065, C3M, Team 6, Nice, France; University of Hawaii at Manoa

**Keywords:** *Aeromonas*, antibiotic resistance, ciprofloxacin, leech therapy, genomics, microbiome

## Abstract

Fluoroquinolones (FQs) and ciprofloxacin (Cp) are important antimicrobials that pollute the environment in trace amounts. Although Cp has been recommended as prophylaxis for patients undergoing leech therapy to prevent infections by the leech gut symbiont *Aeromonas*, a puzzling rise in Cp-resistant (Cp^r^) *Aeromonas* infections has been reported. We report on the effects of subtherapeutic FQ concentrations on bacteria in an environmental reservoir, the medicinal leech, and describe the presence of multiple antibiotic resistance mutations and a gain-of-function resistance gene. We link the rise of Cp^r^ *Aeromonas* isolates to exposure of the leech microbiota to very low levels of Cp (0.01 to 0.04 µg/ml), <1/100 of the clinical resistance breakpoint for *Aeromonas*. Using competition experiments and comparative genomics of 37 strains, we determined the mechanisms of resistance in clinical and leech-derived *Aeromonas* isolates, traced their origin, and determined that the presence of merely 0.01 µg/ml Cp provides a strong competitive advantage for Cp^r^ strains. Deep-sequencing the Cp^r^-conferring region of *gyrA* enabled tracing of the mutation-harboring *Aeromonas* population in archived gut samples, and an increase in the frequency of the Cp^r^-conferring mutation in 2011 coincides with the initial reports of Cp^r^ *Aeromonas* infections in patients receiving leech therapy.

## IMPORTANCE

The role of subtherapeutic antimicrobial contamination in selecting for resistant strains has received increasing attention and is an important clinical matter. This study describes the relationship of resistant bacteria from the medicinal leech, *Hirudo verbana*, with patient infections following leech therapy. While our results highlight the need for alternative antibiotic therapies, the rise of Cp^r^ bacteria demonstrates the importance of restricting the exposure of animals to antibiotics approved for veterinary use. The shift to a more resistant community and the dispersion of Cp^r^-conferring mechanisms via mobile elements occurred in a natural setting due to the presence of very low levels of fluoroquinolones, revealing the challenges of controlling the spread of antibiotic-resistant bacteria and highlighting the importance of a holistic approach in the management of antibiotic use.

## INTRODUCTION

Antibiotic-resistant pathogens and the clinical infections that they cause are a serious concern for human and animal welfare. Because the overuse of antimicrobials in humans and livestock fuels the selection of resistant bacteria, the importance of environmental contamination with antibiotics is receiving increasing attention (1). Point sources, such as hospital and pharmaceutical industry discharges, can introduce large amounts of antibiotics into the environment. Antimicrobials leach and diffuse into their surrounding environments, resulting in concentration gradients over larger areas (1). While environmental levels of antibiotics may not be sufficient to prevent bacterial growth, these levels can select for and maintain resistant mutants (1). This concept is supported by *in vitro* studies demonstrating that low-level antibiotics can select for genetic markers that confer resistance and contribute to the spread of antibiotic-resistant bacteria (2–4). Increases in environmental antibiotic resistance, especially in food products, are important to the One Health initiative, exemplifying avenues of transmission from environmental bacteria to humans and ones that influence human health (5). While previous work studied the effects of low-level antibiotics in lab-grown bacteria, our knowledge is limited regarding the changes that occur in bacterial populations in their natural setting, for example, the host animal (1). In this study, we investigated these dynamics in the gut of the medicinal leech, *Hirudo verbana*, and used this natural system to understand the role of low levels of antimicrobials in enabling resistant bacteria to persist among sensitive strains in their environment.

Medicinal leeches are administered to patients after tissue reconstructive surgery to increase blood flow by releasing vasodilators and anticoagulants while actively removing blood through the process of bloodletting. This treatment for venous congestion promotes tissue salvage and improves surgical outcomes (6–9). In up to 36% of the applications, bacterial infections can occur at the tissue reconstruction site where leeches are administered, reducing the success of the surgery and potentially resulting in serious systemic consequences (6, 9). The suspected cause of these infections originates with the simple microbial community of the H. verbana digestive tract, which includes the human pathogen *Aeromonas* (10–13). We previously reported culturing exclusively Aeromonas veronii from H. verbana (10), but clinicians have reported primarily recovering Aeromonas hydrophila from infected wounds. As infections associated with leech therapy may progress to septicemia (8), it has become best practice to treat patients prophylactically with the widely used fluoroquinolone (FQ) ciprofloxacin (Cp), which dramatically reduces the incidence of these wound infections (9). Since 2011, infections by Cp-resistant (Cp^r^) A. hydrophila strains were reported in seven patients from the United States, Canada, and France, contributing to concerns of a widespread increase in severe wound infections that lead to poor surgical outcomes, including tissue loss, amputation, and septicemia (8, 14–19) (see Tables S1 and S2 in the supplemental material). *Aeromonas* isolates from wounds of patients who received prophylactic ciprofloxacin therapy have been observed to be highly resistant to Cp (20), although the reason for this resistance is unclear.

10.1128/mBio.01328-18.2 TABLE S1  Description of clinical isolates used in this study. Download TABLE S1, DOCX file, 0.1 MB. Copyright © 2018 Beka et al. 2018 Beka et al. This content is distributed under the terms of the Creative Commons Attribution 4.0 International license.

10.1128/mBio.01328-18.3 TABLE S2  Published case reports of Cp^r^ *Aeromonas* spp. cultured in association with medicinal leech therapy. Download TABLE S2, DOCX file, 0.02 MB. Copyright © 2018 Beka et al. 2018 Beka et al. This content is distributed under the terms of the Creative Commons Attribution 4.0 International license.

Sartor et al. (21) raised the possibility that medicinal leeches were exposed to FQs at the farm where they are raised in France by feeding them on blood derived from FQ-treated poultry. Although this practice could explain the occurrence of resistant *Aeromonas* isolates causing leech therapy-associated infections, no strain comparisons or FQ measurements were reported (21). We were interested in determining whether FQs were present in the leech gut and whether the concentrations detected could account for the rise of a Cp^r^ *Aeromonas* population. Using a combination of comparative genome sequence analysis and high-throughput amplicon sequencing, we determined that Cp^r^ clinical and leech-derived isolates are linked, carry resistance genes, and could be detected in the leech digestive tract. We also determined that very low FQ concentrations in the leech digestive tract could select for and maintain naturally occurring symbiotic *Aeromonas* strains with increased Cp^r^.

## RESULTS

### Establishing a collection of clinical and leech-derived aeromonads.

**FIG 1:**
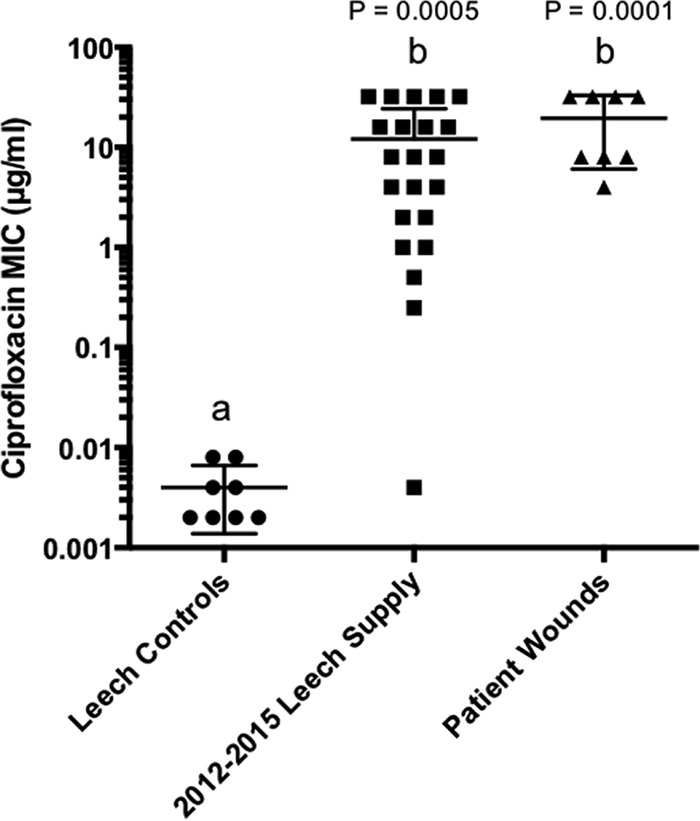
Resistance of *Aeromonas* isolates to Cp. Resistance MICs are plotted for the leech control group, which consists of pre-1999 strains from the supply chain prior to the contamination concern and one strain from a 2012 noncontaminated supplier. MICs are also shown for isolates from the main supply chain and from patients treated with leeches in 2012 to 2015. The *Aeromonas* strains from the main supply chain were significantly more resistant to Cp, based on the Kruskal-Wallis test using Dunn’s multiple-comparison test of sample mean ranks indicated by different lowercase letters. MICs of isolates from pre-1999 leeches differed significantly from those of leech supplies and of patient isolates in 2012 to 2015 (*P* = 0.0005 and <0.0001, respectively).

In order to study the magnitude and prevalence of Cp^r^ among aeromonads, we established a collection of 37 isolates from hirudotherapy wound infections that occurred post-Cp treatment, from leeches obtained in 2012 to 2015 from the FDA-approved supply chain, and from leeches obtained prior to 1999 or from a different supplier (leech control isolates). MIC assays confirmed that wound isolates were Cp^r^ (Fig. 1; also see Table S3 in the supplemental material). MICs of isolates from the FDA-approved supply chain ranged from 0.004 to ≥32 µg/ml with the majority of isolates (77%) being Cp^r^ (≥4 µg/ml) (22). In contrast, the control isolates were all Cp sensitive (Cp^s^), with the observed MICs being far below the cutoff for intermediate Cp resistance (Cp^i^; 2 µg/ml) (22), ranging from 0.002 to 0.008 µg/ml. This suggests that microbes cultured from the digestive tract of leeches from an FDA-approved supplier gained Cp^r^ after 1999 (Fig. 1).

10.1128/mBio.01328-18.4 TABLE S3  Description of isolate sources. Download TABLE S3, DOCX file, 0.01 MB. Copyright © 2018 Beka et al. 2018 Beka et al. This content is distributed under the terms of the Creative Commons Attribution 4.0 International license.

### Detection of FQs inside the leech digestive tract.

Based on the detection of Cp^r^ bacteria inside the leech digestive tract and the suggestion by Sartor et al. that farmed leeches could have been fed FQ-containing blood (21), we wanted to determine if FQs were present inside the leech gut. We analyzed the leech digestive tract content for the presence of two FQs, Cp and enrofloxacin (Ef). Ef is very similar to Cp and is approved for veterinary treatment on poultry farms (1, 23). The digestive tract contents of 10 leeches received in 2014 from the primary FDA-approved supplier were tested using liquid chromatography-mass spectrometry (LC-MS), and Cp was detected in all 10 animals, ranging from 0.01 to 0.04 µg/ml Cp with an average concentration of 0.02 ± 0.007 µg/ml (Table S4). Ef was also detected in most samples (0.01 ± 0.008 µg/ml) but always at a lower concentration than Cp. The presence of Cp in leeches could be due to the deethylation of Ef yielding Cp, which has been shown to occur in the livers of chickens and other animals (24, 25). The detection of Cp and Ef is consistent with the hypothesis that leeches were exposed to FQ-contaminated poultry blood. Alternatively, leeches could have been exposed to FQs through contaminated water or have been directly treated with the antibiotics, although there is no direct evidence supporting these possibilities. The concentration of FQs that we detected in the animals is much lower than the clinical breakpoint for Cp^r^ strains (MIC, ≥4 µg/ml) (22) and only slightly higher than the MIC that we determined for our control isolates (0.002 to 0.008 µg/ml).

10.1128/mBio.01328-18.5 TABLE S4  Detection of fluoroquinolones in leech gut content. Download TABLE S4, DOCX file, 0.01 MB. Copyright © 2018 Beka et al. 2018 Beka et al. This content is distributed under the terms of the Creative Commons Attribution 4.0 International license.

### Competitive growth of *Aeromonas* isolates with or without Cp.

To determine whether FQ concentrations at 1/400 of the resistance breakpoint for *Aeromonas* were sufficient to provide Cp^r^ *Aeromonas* spp. with a growth advantage over a sensitive strain isolated from the leech prior to the concerns regarding FQ contamination, we conducted competition experiments in the leech as previously reported (26) in the presence or absence of Cp. An A. hydrophila strain from a therapy-associated wound infection, CA-13-1, and an A. veronii strain isolated from an FDA-approved medicinal leech, Hv13-B-13b, were highly resistant to Cp (MICs, ≥32 µg/ml) (Table S3). Each strain was competed against the Cp^s^ A. veronii leech-derived control strain, Hm21, which is a well-characterized strain that belongs to the largest phenotypic group of A. veronii leech isolates (10) and competes equally well with other leech isolates in these competition assays (26, 27). The MIC of Hm21 was 0.008 µg/ml as determined with Etests (0.02 µg/ml when grown in broth), and the Cp concentration detected in the leech was as low as 0.01 µg/ml. Based on the Cp sensitivity of Hm21 and the Cp concentrations detected in the leeches, the resistant and susceptible strains were competed in leeches fed 0, 0.0025, 0.007, or 0.01 µg/ml Cp.

**FIG 2:**
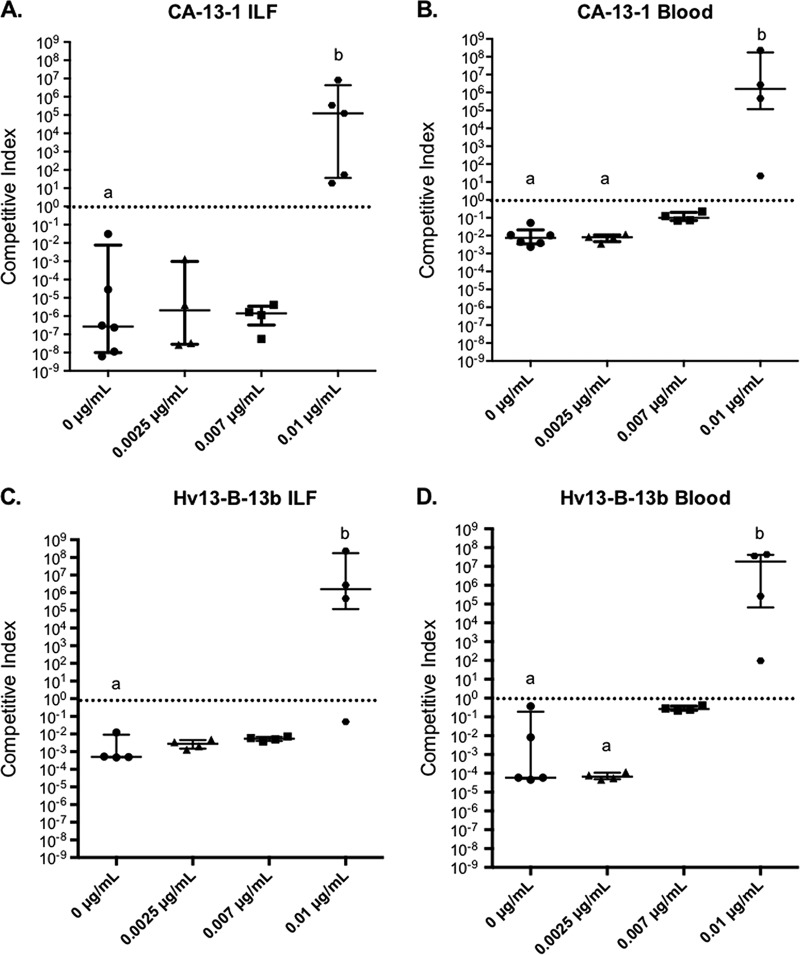
Competitiveness of Cp^**r**^ clinical (CA-13-1) and leech-derived (Hv13-B-13b) *Aeromonas* isolates in the presence and absence of ciprofloxacin. Competitive index (CI) values above 10^0^ indicate that the Cp^r^ strain outcompetes the susceptible pre-1999 leech control isolate, Hm21. (A and C) Leeches from another supplier (in which FQs were not detected) were fed blood meal containing Cp concentrations of 0, 0.0025, 0.007, and 0.01 µg/ml, and intraluminal fluid (ILF) was sampled 72 h postfeeding. (B and D) The same competition assays done *in vitro* (Blood) with the respective conditions. The Cp^s^ strain outcompetes CA-13-1 and Hv13-B-13b in 0.01-µg/ml-Cp-fed leeches and in blood, but this is reversed at lower Cp concentrations. Statistical analyses were performed with the Kruskal-Wallis test using Dunn’s multiple-comparison test, treatment groups which differ significantly from each other (*P* value of <0.05) are indicated with lowercase a and b. Error bars show the median within the interquartile range.

When no Cp or 0.0025 or 0.007 µg/ml Cp was present, the Cp^s^ Hm21 outcompeted CA-13-1 approximately 100,000-fold *in vivo* (Fig. 2). At the same concentrations, Hm21 also outcompeted the Cp^r^ A. veronii strain, although to a lesser extent (~100- to 1,000-fold). The strikingly low colonization ability of the resistant strains in the absence of Cp could indicate a fitness cost of harboring antibiotic resistance markers or that these strains are not as well adapted to the leech digestive tract habitat. Opposite results were obtained in the presence of 0.01 µg/ml Cp, both *in vivo* and *in vitro*. The data confirmed that the Cp concentration detected in the leech digestive tract is sufficient to shift the microbial community toward Cp^r^. The impact of these low FQ levels is reflected in the overall increase in the Cp MICs of *Aeromonas* strains that were cultured from the FDA-approved leeches. Interestingly, only one of 22 isolates had a MIC below 0.1 µg/ml, and the majority exceeded 4 µg/ml. This change in the Cp^r^ could explain the increase of infections in leech therapy patients.

Notably, we also observed a pronounced difference in the competitive indexes between *in vivo* and *in vitro* conditions for one of these strains. The Cp^r^ A. hydrophila strain CA-13-1 had a 4-orders-of-magnitude-lower competitive index *in vivo* than in blood when ≤0.007 µg/ml Cp was present (Fig. 2). These experiments suggest that there are additional colonization barriers that CA-13-1 must overcome *in vivo*, which dramatically lower its ability to compete against the leech isolate Hm21. In contrast to CA-13-1, the competitive indexes of the Cp^r^ A. veronii strain Hv13-B-13b were similar under all four Cp concentrations between the *in vitro* and *in vivo* assays (Fig. 2). This further supports the idea that A. veronii, the dominant symbiont in the leech, is better suited to this niche. In fact, we primarily cultured A. veronii from the H. verbana gut in the past and reported evidence for a high level of horizontal gene transfer between organisms of this group, which are specialized for this particular environment within the medicinal leech (10, 26, 28). The presence of 0.01 µg/ml Cp alleviates the difference between the *in vivo* and *in vitro* results, suggesting a role of the native leech gut microbiota in competing against CA-13-1 (Fig. 2). This effect could be indirect through a modification of a host response, which can ultimately increase the stringency of the competition, or direct, as the presence of other microbes can enhance the competition for nutrients. Importantly, these data show that commonly used *in vitro* fitness assays oversimplify natural conditions and can give dramatically different results. It is likely that the competitive index will be magnified in other host-associated settings and *ex vivo* environments where native microbial communities exert additional *in situ* selective pressures that are absent in the simplified *in vitro* systems. A discrepancy in results obtained from experiments performed under laboratory versus natural conditions affecting resistant bacterial populations has been previously shown in the soybean rhizosphere (29).

### Presence of *gyrA* mutation in *Aeromonas* isolates collected over time.

The competition assay results suggested that the low Cp levels detected in the leech were sufficient to provide a growth advantage to Cp^r^ strains and allow them to outcompete a Cp-susceptible member of the native community isolated in 1996. However, the ratio of Cp^r^ to Cp^s^ *Aeromonas* strains inside the leech digestive tract remains unclear. Because all the leech-derived Cp^r^ strains were isolated using a medium containing Cp, the total *Aeromonas* population in the leech digestive tract could not be quantified. To estimate the abundance of the *Aeromonas* population in the leech digestive tract with elevated Cp^r^, we assessed the frequency of a Cp^r^ indicator mutation in DNA gyrase subunit A, i.e., *gyrA* (S83I). We developed a novel deep-sequencing approach on an Illumina MiSeq allowing the species identification (30) and detecting the characteristic S83I mutation.

**FIG 3:**
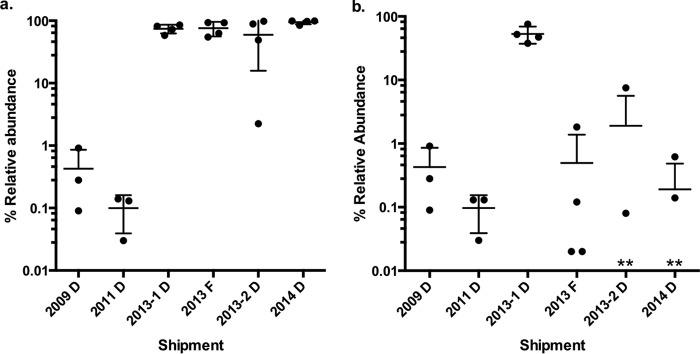
Abundance of the *gyrA* (S83) mutation in leeches over time. Leech crop content was sampled from leeches supplied by the main FDA-approved distributor (D) or farm (F) in 2009, 2011, two shipments in 2013, and one in 2014. We determined the percent relative abundance of *gyrA* (S83I) in total reads (a) and A. hydrophila-specific reads (b). The mean and standard deviation are shown as error bars. An asterisk indicates values for individual leech samples for which there were zero sequencing reads of *gyrA* (S83I).

Deep-sequencing assays across three leech shipments from 2013 and 2014 allowed us to classify 80% of the *gyrA* sequences as originating from A. veronii carrying the Cp^r^-conferring mutation, S83I. In contrast, A. hydrophila *gyrA* (S83I) sequences accounted for between 0.02 and 7.5% of the sequences (Fig. 3). Interestingly, in one additional shipment from 2013 the majority of sequences (45.7 to 74.1% across 4 animals) were identified as A. hydrophila *gyrA* (S83I) (Fig. 3). These data suggest an unexpected variability in the relative abundance of these two species within the leech gut and a disconcerting prevalence of the *gyrA* (S83I) allele over several years. To determine whether a similarly high frequency of the S83I mutation could be detected in *Aeromonas* spp. from digestive tract contents from past shipments, we extended this analysis to include six archived samples obtained in 2009 and 2011. The A. hydrophila *gyrA* (S83I) allele was detected, although this sequence accounted for less than 1% of the total sequences. These findings indicate that A. hydrophila strains carrying the Cp^r^-enabling allele *gyrA* (S83I) have been present at low abundance in leeches sold by medical suppliers for many years and that a dramatic change in the abundance of *gyrA* (S83I)-positive A. veronii occurred since 2011. The relatively low abundance of A. hydrophila in leeches and their frequent recovery from leech therapy-associated wound infections in patient samples raise the interesting possibility that among leech-derived isolates, A. hydrophila strains may be more virulent than A. veronii. However, the identification of these pathogens has been problematic, because A. veronii has been commonly misidentified as A. hydrophila in clinical settings (31).

### Using genomics to establish a link between infection and hirudotherapy.

The observed colonization defect of the clinical A. hydrophila strain in the absence of Cp (Fig. 2) and the higher abundance of A. veronii *gyrA* sequences in the leech digestive tract contents (Fig. 3) point to the possibility that pathogenic A. hydrophila is not well adapted to the leech digestive tract. Because A. hydrophila is commonly found in aquatic environments, it may have been present in the hospital or pharmacy aquaria in which leeches are maintained and then transmitted to the patient via a nosocomial route. In fact, the well-recognized link between hirudotherapy and *Aeromonas* infections is based on a few publications in which biochemical methods were used to identify wound and leech isolates as the same species (15, 18, 32). However, more robust source-tracking of these infectious agents has not been performed. Identifications based on biochemical tests have been shown to misidentify *Aeromonas* species (10, 33). To test the link between hirudotherapy and *Aeromonas* infections, we performed the first genome-based comparison of isolates from hospitals across various geographic locations with those cultured directly from medicinal leeches (Table S1).

**FIG 4:**
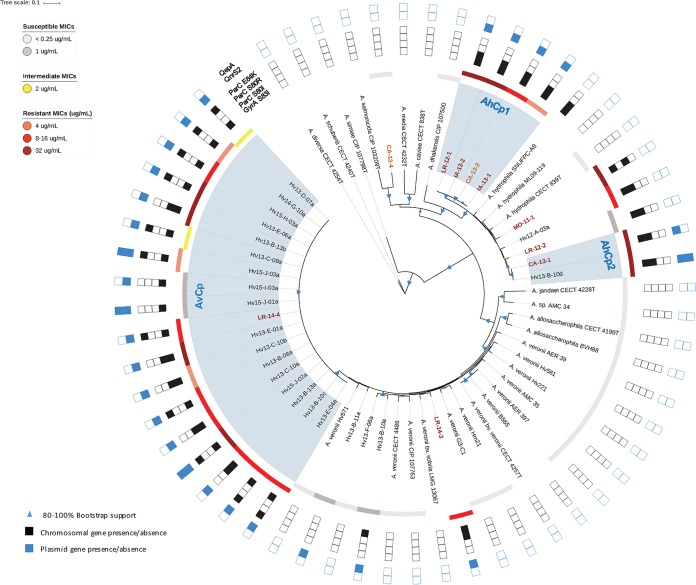
Maximum likelihood reconstruction of 16 single-copy housekeeping genes. Bootstrap support values between 80 and 100% are represented in the tree by variously sized blue triangles (80%, small triangles, to 100%, large triangles). Resistance levels are indicated by colored boxes: highly susceptible, ≤1 µg/ml (light gray); susceptible, 1 µg/ml (dark gray); intermediate, 2 µg/ml (yellow); resistant, 4 µg/ml (orange), 8 to 16 µg/ml (red), and 16 to 32 µg/ml (dark red). The presence of Cp^r^-conferring chromosomal mutations is shown by filled black squares, while the presence of resistance plasmid genes is shown by filled blue squares. For example, MO-11-1 has a black square for ParC^S80r^ to represent that it has an S-80-R mutation. The names of strains derived from clinical isolates are colored in dark red. Names of clinically associated isolates, such as those from leech aquaria, are colored orange.

To accurately identify the 32 clinical and leech-derived isolates, their genomes were sequenced and a suite of housekeeping genes (HKG) was compared with those from 27 published genomes for bioinformatic species identification (Table S5) (34). Using this approach, seven clinical isolates were identified as A. hydrophila, two as A. veronii, and one as a likely novel species (Fig. 4), whereas most isolates were misidentified using biochemical methods (Table S6). In all cases, the HKG-based identifications were further supported by the average nucleotide identity (ANI) analysis (35) (Fig. S1), as each strain’s ANI value was ≥0.96 compared to the type strain for each respective species (Fig. S1). These results are consistent with previous wet-lab studies that identified the majority of wound isolates as A. hydrophila and leech isolates as A. veronii (10, 15–17).

10.1128/mBio.01328-18.1 FIG S1  Average nucleotide identity (ANI) and MLSA distance values. Download FIG S1, DOCX file, 3.9 MB. Copyright © 2018 Beka et al. 2018 Beka et al. This content is distributed under the terms of the Creative Commons Attribution 4.0 International license.

10.1128/mBio.01328-18.6 TABLE S5  Accession numbers and descriptions of clinical and leech isolates’ genomes sequenced in this study. Download TABLE S5, DOCX file, 0.02 MB. Copyright © 2018 Beka et al. 2018 Beka et al. This content is distributed under the terms of the Creative Commons Attribution 4.0 International license.

10.1128/mBio.01328-18.7 TABLE S6  API 20 NE strip phenotypic testing results shown below for *Aeromonas* isolates sequenced in this study. Download TABLE S6, DOCX file, 0.03 MB. Copyright © 2018 Beka et al. 2018 Beka et al. This content is distributed under the terms of the Creative Commons Attribution 4.0 International license.

**FIG 5:**
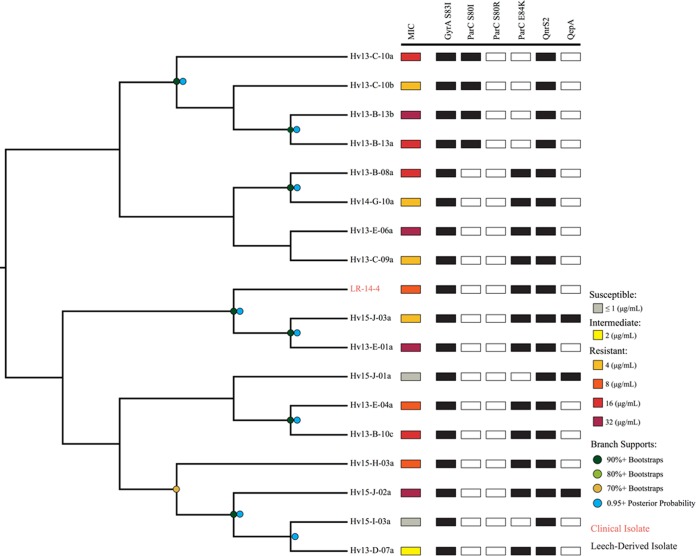
Phylogenetic reconstruction of the AvCp group. This cladogram is a maximum likelihood reconstruction generated from whole-genome alignments. Bootstrap support values are represented by dots: dark green, 90%+ bootstraps; light green, 80%+; khaki, 70%+. Posterior probabilities of 95%+ from a Bayesian inference are represented by blue dots, and branch lengths do not carry meaning. Resistance levels are indicated by colored boxes: susceptible, ≤1 µg/ml (gray); intermediate, 2 µg/ml (yellow); resistant, 4 µg/ml (orange), 8 µg/ml (dark orange), 16 µg/ml (red), and 32 µg/ml (dark red). The presence of Cp^r^-conferring genes and mutations is shown by filled black squares. The names of taxa derived from clinical isolates are colored in orange.

The phylogenetic comparison allowed us to determine the relatedness of clinical and leech isolates by identifying three important clades. Four clinical A. hydrophila strains from the United States and Europe were placed into one clade (AhCp1) and shared identical HKG sequences (29,688 bp) (Fig. 4). The wound isolate, CA-13-1, and leech digestive tract isolate, Hv13-B-10d, were both confirmed to be A. hydrophila and also had identical HKG sequences (AhCp2). Notably, we identified 18 A. veronii strains that had nearly identical HKG sequences and were grouped into a single clade comprising 17 leech isolates and strain LR-14-4, a leech therapy wound isolate. The HKG sequences of these isolates differed by 0 to 8 bp (median, 1 bp) (AvCp). The AvCp clade was further analyzed by performing a whole-genome alignment and by calculating a well-supported phylogeny that grouped the clinical isolate LR-14-4 within a highly supported clade containing two leech-derived isolates (Fig. 5). Although the genetic content was nearly identical, the Cp MICs of the AvCp group varied greatly: 14 isolates were Cp^r^, two were Cp^i^, and two were Cp^s^ (Table S7). Nearly isogenic strains that span the scope of resistance phenotypes have rarely been reported (1) and may help in the elucidation of additional resistance mechanisms.

10.1128/mBio.01328-18.8 TABLE S7  MICs of ciprofloxacin for all *Aeromonas* isolates included in Fig. 1. Download TABLE S7, DOCX file, 0.01 MB. Copyright © 2018 Beka et al. 2018 Beka et al. This content is distributed under the terms of the Creative Commons Attribution 4.0 International license.

ANI analysis of the AhCp1, AhCp2, and AvCp clades revealed that the genomes of every strain within each clade were extremely similar to each other (99.89% identity or higher), although none were identical (Fig. S1). In contrast, the intraspecies ANI values for all Cp^s^ leech isolates were much lower (median of 96.24% identity), a finding that agrees with the results of the HKG phylogeny, in which the Cp^s^ leech isolates did not form a monophyletic group.

Both the ANI and HKG analyses reveal a very close relationship among the clinical strains and those cultured directly from leeches. The two strains from AhCp2 had ANI values of 99.97%, and LR-14-4 grouped with two leech isolates with which it shared an average ANI value of 99.87%, confirming the leech-to-human transmission of the Cp^r^ clinical isolates. In these three clades, the high similarity of the genomes of strains with elevated resistance to Cp is consistent with the presence of a strong selection pressure promoting the proliferation of Cp^r^ *Aeromonas* strains in the leech gut.

### Whole genomes provide insight into the resistance mechanisms of the *Aeromonas* isolates.

Further analyses of the genomes allowed us to gain insight into the genetic factors underlying Cp resistance. While the first step to becoming resistant to Cp typically begins with the acquisition of the *gyrA* (S83I) mutation, greater resistance can be attained by additional point mutations in topoisomerase IV (*parC*), e.g., *parC* (E84K) or *parC* (S80I) (20, 36). The acquisition of resistance genes, such as efflux pumps encoded by *qepA*, the *qnr* (quinolone resistance) gene family that protects DNA gyrase, and acetylases such as *aac(6′)-Ib-cr*, leads to further resistance (20, 37–40), resulting in synergistic increases of the MIC (41). The mutation *gyrA* (S83I) was present in all Cp^r^ and Cp^i^ strains, as well as in four Cp^s^ strains (MICs, 0.25, 0.5, 1, and 1 µg/ml Cp) that had at least an ~60-fold-higher MIC than the average MIC (0.0044 µg/ml) of our control strains (Table S7). These results confirm that the *gyrA* (S83I) mutation can be used as a sentinel marker for bacteria with an elevated MIC, as it was used to estimate the size of the population with elevated resistance in the deep-sequencing experiment (Fig. 3).

We wondered if a similar stepwise pattern of Cp^r^ acquisition occurred in our *Aeromonas* strains, and the AvCp clade provided us with the opportunity to address this question. The stepwise acquisition of resistance genes appears to have independently occurred multiple times within the AvCp clade, since all of the strains have the *gyrA* (S83I) mutation and carry *qnrS2* but the two sensitive strains do not have a mutation in *parC* (Fig. 5). We identified two clades (each containing four strains) with significant bootstrap support that carry the *parC* (E84K) allele and two clades (each consisting of two strains) that carry the *parC* (S80I) allele, suggesting that these *parC* mutations were acquired independently multiple times after the *gyrA* S83I mutation (Fig. 5). This is evidence that the stepwise acquisition of mutations observed in laboratory experiments also occurs in the environment.

The acquisition of plasmids carrying *qnr* genes and efflux pump-encoding genes is an important factor for further elevating the fluoroquinolone resistance level. The *qnr* genes have been identified on plasmids of various incompatibility (Inc) groups and sizes that exist within a wide range of hosts (40), and the observations are consistent with our findings. Every Cp^r^ A. veronii strain carried *qnrS2* on an ~34.5-kb conjugative plasmid belonging to the IncU group. These plasmids are very similar to pAS37, which has been shown to carry *qnrS2* in Aeromonas caviae (42). All Cp^r^ A. hydrophila wound isolates contained *gyrA* (S83I), either *parC* (S80I) or *parC* (E84K), and *qnrS2*, except for strain MO-11-1, which lacked *qnrS2* (Fig. 4). The predicted QnrS2 proteins were all 218 amino acids (aa) in length and identical across the strains analyzed in our study. CA-13-1 and Hv13-B-13b, which were competed in the leech digestive tract against the sensitive control (Fig. 2), both carry chromosomal mutations and the *qnrS2* gene on an IncU plasmid. *qnrS2* was carried on a large IncU plasmid, except in A. hydrophila strain Hv13-B-10d, where it was located on a small (~6.8-kb) high-copy-number plasmid, pHv13-B-10d-C. The spread of *qnrS2* was likely due to a transposition event as *qnrS2* is flanked by a 22-bp inverted repeat and a 5-bp duplication, suggesting that it is located on a mobilizable element that lacks a transposase gene. This transposition is similar to what has been reported for *qnrS2* in A. caviae (42). Whether or not the presence of high-copy-number plasmids leads to elevated QnrS2 levels remains to be evaluated in *Aeromonas*, but studies of other genera support the idea that the presence of *qnrS2* on plasmids does facilitate higher levels of Cp^r^, even if the production of QnrS2 does not significantly augment the MIC (40). Hv13-B-10d and two Cp^r^ A. veronii strains also carried *qepA*, a presumptive quinolone efflux pump, on a small plasmid. One particularly interesting strain is A. veronii Hv13-B-11a, which carries *qnrS2* on the IncU plasmid, pHv13-B-11a-A, but has no mutation in *gyrA* or *parC*. This strain is Cp^s^ but has a much higher MIC (1 µg/ml) than the susceptible control strains. These data indicate that the IncU plasmid carrying *qnrS2* is transferred readily between *Aeromonas* species and that resistance plasmids are maintained even in Cp^s^ strains that do not harbor chromosomal mutations known to facilitate Cp^r^.

## DISCUSSION

In this study, we describe the rise of FQ resistance in a natural symbiont of the medicinal leech digestive tract. Our results indicate that an FQ concentration of 0.01 µg/ml within the leech is sufficient to ensure the long-term persistence of Cp^r^ *Aeromonas* spp. and likely promotes the acquisition and spread of antibiotic resistance genes. The observed rise in the abundance of *Aeromonas* strains carrying a resistance-conferring *gyrA* mutation (Fig. 3) and the wide distribution of a plasmid carrying *qnrS2* among both A. veronii and A. hydrophila strains (Fig. 4) suggest that FQ concentrations as low as 0.01 µg/ml impose a sufficient selective pressure for the maintenance of resistance markers. Our data show that in *Aeromonas* isolates exposed to low amounts of Cp, the mutation in *gyrA* likely occurred before a mutation in *parC*. However, given that the *gyrA* (S83I) mutation is thought to be a first step toward resistance, it is notable that a single strain that did not have the *gyrA* or *parC* mutation, Hv13-B-11a, acquired the *qnrS2*-carrying plasmid, contrary to the canonical paradigm (20). This observation is of particular importance as conjugatable resistance plasmids can be transferred at very high frequencies, which can surpass mutation rates in a given gene under certain conditions, e.g., the IncU plasmid transfer rate from Aeromonas salmonicida into Escherichia coli is high compared to an S83L mutation in *gyrA* in clinical E. coli strains (43–45).

Our results strongly suggest that the therapeutic use of leeches containing FQs led to nosocomial infections by Cp^r^ strains following leech therapy. For a safe and effective therapeutic administration of leeches, a monitoring program for FQ levels and Cp^r^ strains should be implemented by the suppliers, and alternative antibiotic therapies against leech-acquired *Aeromonas* infections are needed (15, 18). The source of sub-MIC levels of FQs within the medicinal leech must be identified and eliminated, although the threat of resistant *Aeromonas* from contaminated environments remains. For example, Cp^r^ *Aeromonas* strains have been cultured from environmental sources, including lakes, rivers, and sewage treatment facilities (46–49), and environmental strains have been observed to carry IncU plasmids harboring *qnrS2* (42). If such strains are present in the water that is used to ship leeches to hospitals or pharmacies, or if these strains colonize the leech surface or digestive tract, they might lead to nosocomial infections.

The *in vivo* fitness experiments in the presence of 0.01 µg/ml Cp demonstrate the ability of the Cp^r^ strains to outcompete a natural leech symbiont that is representative of the *Aeromonas* community prior to the FQ contamination. Interestingly, even susceptible strains derived from leeches between 2012 and 2014 displayed elevated MICs, and this evolution of resistance in the *Aeromonas* population occurred outside a lab environment. This suggests that very small amounts of Cp in animal digestive tracts and perhaps other environments are sufficient to select for strains with elevated resistance. Our *in vivo* results are supported by previous experiments conducted *in vitro* that emphasized the importance of sub-MICs of antimicrobials (4), as they induced predictable changes in the microbial community. In chemostats inoculated with human fecal matter containing microbiota exposed to various Cp levels, a concentration as low as 0.43 µg/ml was enough for enterococci to incur a loss of colonization resistance to a pathogenic *Salmonella* strain, while lower Cp concentrations did not affect colonization resistance (50). Sub-MIC Cp levels were observed to promote biofilm formation of a respiratory tract pathogen, Moraxella catarrhalis, under anaerobic conditions (51). In the early 2000s, the increased occurrence of human infections due to FQ^r^ *Campylobacter* spp. was linked to the ingestion of poultry meat from chickens that were treated with an FQ and whose meat became contaminated with resistant bacteria (52). More recently, there were significant increases in the number of FQ^r^ *Campylobacter* isolates from surveyed farms compared to the averages collected between 2004 and 2008 (53). The inclusion of *in vitro* and *in vivo* competition assays in our study revealed important differences between laboratory and natural settings and highlights shortcomings of laboratory experiments that cannot be ignored. We hypothesize that the complexity of microbial communities in the environment and the resulting increased competition for resources may intensify the consequences of low-level antimicrobials and explain discrepancies between *in vitro* and *in situ* assays that have been observed previously (29).

Naturally occurring isogenic strains isolated from the environment are very uncommon (1), and their analysis provides researchers with an advantage in studying evolutionary relationships and resistance mechanisms. The AvCp clade strains were obtained over a period of 3 years, and although they are not identical, their high similarity suggests that leech husbandry has indirectly facilitated the rise of very similar strains with elevated resistance to Cp. The 18 AvCp A. veronii strains likely arose from a common ancestor that acquired the key *gyrA* (S83I) mutation, and the descendants contained an average of 679 single nucleotide polymorphisms (SNPs) (ranging from 352 to 1,163, with a median of 649) across their ~4.8-Mbp genomes. The high degree of similarity of these strains provides a rare opportunity to analyze the factors that lead to increased Cp resistance levels. The results of the whole-genome analysis suggest that the acquisition of resistance markers (mutations in *parC* and the gain of *qepA*) occurred independently multiple times in a stepwise manner (Fig. 3). Despite this high level of similarity and possession of *qnrS2*, *gyrA*, and *parC* mutations, this clade ranged from being susceptible to being highly resistant to Cp (MICs of 0.25 to >32 µg/ml Cp) (Fig. 4; see also Table S3 in the supplemental material). Currently, we cannot account for all of the differences in MICs of the individual AvCp isolates. Possible additional factors include differences in expression levels of *qnrS2*, mutations that affect cell envelope permeability, and yet-to-be-identified resistance mechanisms (38, 54, 55).

Our study also points to the changes that have occurred in the leech digestive tract microbiota over time. The amplicon sequencing analysis of the *gyrA* gene revealed a significant rise in the relative abundance of *Aeromonas* species with the Cp^r^-related *gyrA* mutations between 2011 and 2013 (Fig. 5). The competition data suggest that these elevated-Cp^r^ strains, which appear to dominate the gut, can outcompete the typical *Aeromonas* strain (Hm21) only in the presence of FQ contamination. As with Hm21, which represents the *Aeromonas* community prior to FQ contamination, other *Aeromonas* strains isolated prior to this problem also lacked the *gyrA* and *parC* mutations and plasmid resistance genes (Fig. 4). It is very likely that these changes to the *Aeromonas* community in the leech gut were due to the coincidental exposure of FQ either as a direct consequence of feeding blood derived from FQ-treated poultry or through other means of FQ contamination at the leech farm. The potency of FQ is alarming, and whatever the source of contamination may be, low-level antimicrobials in a natural environment appear to facilitate the spread of resistance markers (1).

Overprescription and incorrect usage of antimicrobials in agriculture have played a major role in the rise of antibiotic resistance, and recent literature has emphasized that the excessive application of antibiotics on farms can result in the indirect contamination of soil, rivers, and animal food products (1, 56). A large fraction of antibiotics released into the environment is in an active form, unmetabolized by animal renal or digestive systems (57). Studies on pasture animals observed that FQs are excreted mostly unchanged and thus can be found in farm soil (58). Discharge from pharmaceutical plant and hospital efflux systems further exacerbates antibiotic pollution of these natural sites (1, 52). Antimicrobials emanate from these point sources and form spatial and temporal concentration gradients that encompass the concentrations used in our competition experiments.

Interestingly, some studies have quantitated the concentrations of FQs in various environments. Several studies have reported the presence of FQs at concentrations similar to those detected in our study, e.g., in animal farm wastewater (up to 0.0075 µg/ml Cp), river water (up to 0.0059 µg/ml Cp), and manure (up to 19 mg/kg Ef) (59, 60). One study investigating the effluent of a sewage treatment plant near several pharmaceutical plants reported Cp levels ranging between 28,000 and 31,000 µg/liter and in a follow-up study reported contamination of well water with antibiotics in the surrounding area (61, 62). A meta-analysis of the occurrence and sources of FQs in the environment showed that an average of 0.021 µg/ml Cp occurs in hospital wastewater (63), while in another study a median concentration of 0.163 µg/ml Cp and values as high as 6 µg/ml were also detected in water (64). The detection of Cp in water is especially important because it is resistant to degradation in aqueous environments and can remain biologically active for long periods of time (64). The same study found evidence for the increased occurrence of Cp resistance genes in soil with prolonged exposure to below-therapeutic levels of Cp (64). While concerns have been raised regarding the toxicological risks associated with pollutant antibiotics, the effects on the rise and spread of resistance mechanisms in bacterial communities in natural environments have drawn relatively little attention (1, 63). When considering the environmental impact of FQs in the future, it is important to assess the influence of very low antibiotic levels on the gain, spread, and persistence of antibiotic resistance genes and mutations in environmental bacteria.

Our study demonstrates that very low levels of antibiotics exert a selection pressure on microbial populations and that *in vitro* experiments can underestimate the effects of antibiotics in the natural environment. Antimicrobial concentrations well below the clinical breakpoint can lead to a dramatic increase in the abundance of strains with elevated resistance and the spread of plasmid-carried resistance genes between different species culminating in an ecological disturbance. If FQ pollution is not better controlled, FQ^r^ strains will replace sensitive ones and spread resistance to pathogens, leading to an adverse effect on the wellbeing of humans, as postulated by the One Health Initiative (65).

## MATERIALS AND METHODS

### Strains and growth conditions.

Clinical strains were provided by the UCLA School of Medicine, University of Iowa Hospitals and Clinics, Washington University in St. Louis, and the University Hospital of Montpellier (France). Strains were isolated from the following settings: wounds of patients who received leech therapy, surgical instruments used on one of these patients, or aquarium tanks in which the leeches used on patients were housed (see Table S1 in the supplemental material).

The leech-derived strains used in this study were isolated from leeches obtained from various shipments from the main FDA-approved distributor, Leeches USA, Westbury, NY, USA; the FDA-approved leech farm Ricarimpex SAS, Eysines, France; and another distributor and leech farm in Europe, Biebertaler Blutegelzucht, Biebertal, Germany. Leeches from the main FDA-approved distributor were shipped in December 2012, February 2013, April 2013, June 2013, and November 2014. Leeches were dissected as previously described (10). Briefly, leeches were anesthetized in 70% ethanol and dorsally dissected through the crop of the digestive tract. Sterile swabs were used to collect the intraluminal fluid (ILF) from the leech crop and were initially streaked onto LB medium plates with and without Cp-HCl (Santa Cruz Biotechnology Inc., Dallas, TX). Before 2012, control *Aeromonas* isolates were cultured onto LB medium containing no Cp. For culturing *Aeromonas* isolates from leeches after 2012, Cp was present in plates at the following concentrations: 0 µg/ml, 1 µg/ml and/or 2 µg/ml, and 4 µg/ml and/or 6 µg/ml. Plates were then incubated at 30°C for approximately 14 h to identify naturally resistant bacterial subpopulations. Strains were subcultured onto plates with the same ciprofloxacin concentrations as plates on which they were first isolated and preserved as frozen stock.

### DNA extraction, genome library preparation, and sequencing.

Genomic DNA was extracted from pure bacterial cultures using the Epicentre MasterPure Complete DNA and RNA purification kit (Epicentre, Madison, WI) per the manufacturer’s instructions. Genomic DNA was then quantified using a Qubit 2.0 fluorometer (Life Technologies, Inc., Carlsbad, CA) and diluted to 0.2 ng/µl for Illumina Nextera XT (FC-131-1096) (Illumina, Inc., San Diego, CA) DNA library preparation. Genomic tagmentation, PCR of tagged DNA, and PCR product cleanup were done according to the manufacturer’s instructions. The Qubit 2.0 fluorometer and the Agilent 2100 Bioanalyzer (Agilent Technologies, Santa Clara, CA) with the high-sensitivity DNA kit were both used for library dilution to 4 nM for loading into an Illumina MiSeq sequencer, which generated 250-bp reads. Demultiplexing was performed as previously described (13). Leech control isolate Hm21 was sequenced in a different study as previously described (10), and leech-derived isolate Hv13-B-10d was sequenced using PacBio RS II.

### Quality filtering of reads, genome assembly, and annotation.

For Illumina-sequenced strains, paired MiSeq fastq read 1 and read 2 files were imported into CLC Genomics Workbench software (CLC Bio-Qiagen, Aarhus, Denmark). Reads were trimmed, and those with *Q* scores of ≥15 were kept for the downstream assembly. Genome *de novo* assemblies were done with scaffolding using the same software; gene prediction and annotation of assembled genomes were performed using Prokka (66) for all strains except for the five leech control isolates, which were annotated using the RAST server (67) (Tables S3 and S5).

### Species identification and resistance marker detection by sequencing.

Illumina-compatible amplicon primers (Table S8) were designed that amplify a 383-bp region of DNA gyrase subunit A, *gyrA*, covering the region of amino acid positions 63 to 176, based on the TruSeq amplicon format previously described by Nelson et al. for the analysis of 16S rRNA amplicons (68). We determined this fragment of *gyrA* to be sufficient for *Aeromonas* species discrimination, and the amplified region includes the nucleotide positions corresponding to a known point mutation leading to an amino acid substitution (S83I) that is thought to be the first step in acquiring resistance (36). Bioinformatic analysis of these amplicons from multiple leech samples allowed us to determine both relative *Aeromonas* species abundance and the presence of mutations that can potentially confer resistance to ciprofloxacin and enrofloxacin.

10.1128/mBio.01328-18.9 TABLE S8  Primer sequences for Illumina *gyrA* deep sequencing. Download TABLE S8, DOCX file, 0.01 MB. Copyright © 2018 Beka et al. 2018 Beka et al. This content is distributed under the terms of the Creative Commons Attribution 4.0 International license.

*gyrA* amplicons for high-throughput sequencing were generated in triplicate PCRs using the fusion primers (sequences provided in Table S8) with the method described for 16S rRNA gene amplicons described by Nelson et al. (68). The reaction mixtures included 12.5 µl Phusion high-fidelity PCR master mix with 25 μl HF buffer, 3 µM forward and reverse primer, 20 ng DNA template, and distilled water (dH_2_O) to final volume. The PCR cycling conditions were 95°C for 5 min, followed by 30 cycles of 95°C for 30 s, 55°C for 30 s, and then 72°C for 1.5 min followed by a final 72°C for 5 to 10 min before cooling to 4°C. The amplified products were pooled and checked by agarose gel electrophoresis before purification with an 0.65× volume of AMPure XP beads. The purified amplicons were quantified by PicoGreen and sized on an Agilent 2100 Bioanalyzer with the high-sensitivity DNA kit to pool equimolar amounts from each sample to form the final sequencing library.

Sequencing was performed on an Illumina MiSeq using a 2- by 250-bp paired-end protocol. After sequencing, the reads were demultiplexed according to their sample indexes, and read pairs were merged using SeqPrep to form single, high-quality contigs which were then quality trimmed using a *Q*_30_ cutoff over a 10-bp sliding window and minimum length cutoff of 375 bp. The primer sequences were removed with Cutadapt (https://cutadapt.readthedocs.io/en/stable/), and the reads were then formatted for analysis using QIIME.

For QIIME analysis, the reads from all samples were clustered by Uclust based on 99.5% sequence identity, which allows for less than a 2-bp difference between reads. Representative sequences were then selected and aligned against a set of reference *gyrA* sequences selected from *Aeromonas* strains for which the gyrase A sequence was available in GenBank, including sequences from isolates whose genomes have been fully sequenced. Clusters for which two or fewer sequences were clustered were removed from further analysis to limit the effect of spurious sequences due to PCR and sequencing errors. Species-level taxonomic assignments were made to the representative sequences using BLAST against the known reference sequences. The presence of potential antibiotic resistance-conferring mutations was determined using a custom perl script.

### MIC determination of all isolates sequenced in this study.

In accordance with the Clinical and Laboratory Standards Institute (CLSI) guidelines, strains were first grown on blood agar (BA) and incubated for 14 h at 30°C. To prepare the inoculum, isolated colonies from the BA plates were suspended in 0.85% NaCl solution to the equivalent of an 0.5 McFarland turbidity standard. Mueller-Hinton agar (MHA) test plates were inoculated with the cultures, and Etest strips (BioMérieux, SA) with an analytical range from 0.002 to ≥32 µg/ml were applied to MHA to determine MICs. MICs were interpreted after 16 to 18 h of incubation at 35°C per manufacturer’s instructions (document 16246A, BioMérieux) using CLSI interpretation criteria (22) for most of the *Aeromonas* isolates in Fig. 4 (Table S7). The criteria state that *Aeromonas* isolates are Cp^s^ if they are inhibited by ≤1 µg/ml Cp, are Cp^i^ if the MIC is 2 µg/ml Cp, and are Cp^r^ if the MIC is ≥4 µg/ml.

### Competition assays.

Competition assays were conducted as described previously (69). Briefly, leeches were fed heat-inactivated sheep’s blood (Quad 5, Ryegate, MT) inoculated with 500 CFU/ml each of a test strain and a competitor strain to assess the colonization capability of the test strain *in vivo*. The competitor strain for all assays was Hm21RT, a spontaneous rifampin-resistant mutant containing a trimethoprim cassette inserted into the chromosome via a mini-Tn*7* (70). This competitor strain was derived from the leech strain Hm21, which was isolated from the crop of the medicinal leech, *Hirudo verbana* (10). Test strains were ciprofloxacin-resistant *Aeromonas* leech isolates that were also selected for spontaneous rifampin resistance. Growth rates of all strains were determined in LB at 30°C in order to confirm that the mutants did not exhibit any growth defects *in vitro*. Hm21RT was also competed against another commonly used competitor strain, Hm21RS, a spontaneous rifampin- and streptomycin-resistant mutant of Hm21, to verify that Hm21RT’s growth in the leech did not vary from Hm21RS (data not shown). The following final concentrations of ciprofloxacin were also used in the blood meals: 0, 0.0025, 0.007, and 0.01 µg/ml. Leeches used were obtained from BBEZ (Bierbertaler Blutegelzucht GmbH, Bierbertal, Germany). At least four animals were used for each competition, kept at 25°C after feeding, and assayed at 72 h. This time point was chosen because growth of A. veronii has already plateaued inside the leech gut, thereby minimizing the effects of small differences in growth rate between the test and competitor strains (27).

Competition indexes (CIs) were calculated as follows: (test strain_output_/competitor strain_output_)/(test strain_input_/competitor strain_input_). A CI of 1 indicated that the test strain colonized to the same levels as the competitor strain, whereas a CI of <1 indicated that the test strain had a colonization defect. The limit of detection was 10 CFU/ml.

### Growth in blood.

Prior to feeding, an aliquot of each heat-inactivated blood meal inoculated with the competitor and test strains was removed and incubated for 72 h at 25°C. Samples were serially diluted and plated as described above for the *in vivo* competition assay.

### Statistical analysis.

Data were analyzed in GraphPad Prism 6 (GraphPad, San Diego, CA). A Kruskal-Wallis one-way analysis of variance with Dunn’s *post hoc* test was used to determine if the CIs differed from one another (*P* < 0.05). Sample means in Fig. 2 were log transformed due to high variation and analyzed with a one-sample *t* test to determine if sample means were significantly different (95% confidence interval) from a CI value of 1.

### Detection of ciprofloxacin and enrofloxacin using HPLC.

Compounds were quantified via ultrahigh-performance liquid chromatography electrospray ionization mass spectrometry (UHPLC-ESI-MS) with accurate-mass detection. HPLC was performed with a reverse-phase HPLC column: an Agilent PLRP-S PSDVB column with 3.0-μm particles and dimensions of 50 mm in length and 1.0 mm in diameter (P/N PL1312-1300) was used with an Agilent 1290 HPLC system. The column was maintained at 50°C with a flow rate of 0.6 ml/min. Chromatography was as follows: solvent consisted of water with 0.1% (vol/vol) formic acid for channel A and acetonitrile with 0.1% formic acid for channel B. Following column equilibration at 5% B, the sample was injected via autosampler, and the column was flushed for 1.0 min. From 1.0 min to the end of the run, the column eluant was directed to the MS source. From 1.0 min to 4.0 min, the gradient was linearly ramped from 5% to 95% solvent B. From 4.0 to 4.8 min, the column was held at 95% B, and from 4.8 to 5.0 min, the column was reequilibrated with 5% solvent B. Ciprofloxacin eluted starting at 2.7 min, and enrofloxacin eluted starting at 2.75 min.

The mass spectrometer used was an Agilent 6538 quadrupole time of flight (QTOF) spectrometer with ESI source; resolution is approximately 20,000 and accuracy is 1 ppm. Source parameters were as follows: drying gas, 8.0 liters/min; drying gas heat at 350°C; nebulizer, 55 lb/in^2^; capillary voltage, 3,500 V; capillary exit, 100 V. Spectra were collected in positive mode as appropriate from 50 to 1,700 *m/z* at a rate of 2 Hz.

Samples were quantified with the Agilent MassHunter Quantitative Analysis package, using centroid data mode and peak definitions of 332.1377 and 360.1687 *m/z* for ciprofloxacin and enrofloxacin, respectively. Both analytes used a ±50-ppm window for the *m/z* definition, which was evaluated for lack of interfering background signals with the samples. The relative standard deviation was determined using error propagation from the curve fit and technical replicates.

Blanks were run between each sample to eliminate the possibility of carryover interferences, and external standard curves were conducted with authentic ciprofloxacin and enrofloxacin standards (Sigma-Aldrich, Poole, United Kingdom).

### MLSA reference tree generation.

The multilocus sequence analysis (MLSA) reference tree in Fig. 4 was generated using the method described in the work of Colston et al. (34). Sixteen housekeeping genes (*atpD*, *dnaJ*, *dnaK*, *dnaX*, *gltA*, *groL*, *gyrA*, *gyrB*, *metG*, *mdh*, *radA*, *recA*, *rpoC*, *rpoD*, *tsf*, and *zipA*) were used for the MLSA. The full-length sequence of each gene was initially derived from the previously published genome of A. veronii Hm21, and these sequences served as queries for BLAST searches against the annotated proteins of all 56 genomes. Multiple sequence alignments (MSAs) were generated by aligning the genes using MUSCLE (v3.8.31) (71). In-house scripts created a concatenated alignment of all 16 genes. A model of evolution was determined by using the Akaike information criterion with correction for small sample size (AICc), as implemented in jModelTest 2.1.4 (72). A maximum likelihood (ML) phylogeny was generated from the concatenated MSA, and individual gene phylogenies from the individual gene MSAs were determined by using PhyML (v3.0_360-500M) (73). PhyML parameters consisted of a general time-reversible (GTR) model, estimated proportion of invariable sites (p-invar), 4 substitution rate categories, estimated gamma distribution, and subtree pruning and regrafting enabled with 100 bootstrap replicates.

### MLSA and genome alignment distance calculation.

The number of differences between the sequences in the concatenated alignment was calculated using the R package Ape. The dna.dist function was called with the model parameter set to “N” and pairwise.deletion parameter set to “TRUE.”

### Average nucleotide identity analysis.

Assembled contigs were reconstituted from the RAST-generated GenBank files for all genomes by using the seqret function of the EMBOSS package (74). All genomes were treated in the same manner to ensure that any biases were consistent across the entire data set. JSpecies1.2.1 (75) was used to analyze these contig sets for the ANI, using default parameters. We report here the averages of the reciprocal comparisons.

### Whole-genome phylogenetic reconstruction.

A whole-genome alignment of the 18 members of the AvCp clade was generated from sequence files in GenBank format using the progressiveMauve algorithm of Mauve (76). Hm21, Hv221, Hv571, Hv13-B-10a, and Hv13-B-11a were included to serve as outgroups for rooting purposes. The XMFA alignment files were converted into FASTA format using in-house scripts. A model of evolution was determined by using the Akaike information criterion with correction for small sample size (AICc), as implemented in jModelTest 2.1.4 (72). Phylogenies were calculated using both RAxML v8.1.17 (77) under GTR CAT and GTR plus estimated gamma plus invariable sites models (producing identical topologies) and MrBayes v3.2.4 x64 (78) under a GTR model, with estimated gamma.

### Data availability.

All scripts used for analysis, along with the *gyrA* reference data sets, are available from http://github.com/joerggraflab/SARIS.

### Accession numbers.

All *gyrA* sequencing data were deposited under BioProject PRJNA296880 to the INSDC SRA. The URL is https://www.ebi.ac.uk/ena/data/view/PRJNA296880. All new genome sequencing data presented in this study have been deposited to the European Nucleotide Archive under the study accession number PRJNA297409, except for one genome which is deposited under the study accession number PRJEB6940. The URL is https://www.ebi.ac.uk/ena/data/view/PRJNA297409. More information regarding sequencing metadata and individual sample accession numbers is shown in Table S5.
