# Supplementary material for: Low-Level Antimicrobials in the Medicinal Leech Select for Resistant Pathogens That Spread to Patients
Source: mBio. 2018 Jul 24;9(4):e01328-18. doi: 10.1128/mBio.01328-18 (PMC6058295; doi:10.1128/mBio.01328-18)
Supplement: TABLE S1 [file mbo004183985st1.docx]

**Supplementary Table 1. Description of clinical isolates used in this study.**

| \| Source \| Published Name \| Strain Name \| Year Isolated or Published \| *Aeromonas* spp. \| Description \| CpR *^a^* \| Reference \| \| --- \| --- \| --- \| --- \| --- \| --- \| --- \| --- \| \| California, USA \| AH1 \| CA-13-1 \| 2013 \| *A. hydrophila* \| 9yr old patient's face wound; mandibular surgery \| R \| Giltner *et al.,* 2013 \| \|  \| AH2 \| CA-13-2 \| 2013 \| *A. hydrophila* \| distraction arm of surgical instrument used on patient \| R \| \|  \| AH4 \| CA-13-4 \| 2013 \| *Aeromonas* sp. \| leech aquarium water \| S \| \| Iowa, USA \|  \| IA-13-1 \| 2013 \| *A. hydrophila* \| otolaryngeal :microvascular free flap \| R \| n/a \| \|  \|  \| IA-13-2 \| 2013 \| *A. hydrophila* \| otolaryngology: microvascular free flap \| R \| \| Languedoc-Roussillon, France \|  \| LR-12-1 \| 2012 \| *A. hydrophila* \| cancer patient scalp infection in  May 2012 \| R \|  \| \|  \|  \| LR-12-2 \| 2012 \| *A. hydrophila* \| isolated from aquarium in which leech carrying LR-12-2 was kept \| R \| n/a \| \|  \|  \| LR-14-3 \| 2014 \| *A. veronii* \| skin lesion isolate from Dec 2014 \| R \|  \| \|  \|  \| LR-14-4 \| 2014 \| *A. veronii* \| skin lesion isolate from Dec 2014 \| R \|  \| \| Missouri, USA \| *A. hydrophila* 07-345-3437 \| MO-11-1 \| 2011 \| *A. hydrophila* \| leech therapy wound isolate \| R \| Wang *et al.,* 2011 \| |
| --- | --- | --- | --- | --- | --- | --- | --- | --- | --- | --- | --- | --- | --- | --- | --- | --- | --- | --- | --- | --- | --- | --- | --- | --- | --- | --- | --- | --- | --- | --- | --- | --- | --- | --- | --- | --- | --- | --- | --- | --- | --- | --- | --- | --- | --- | --- | --- | --- | --- | --- | --- | --- | --- | --- | --- | --- | --- | --- | --- | --- | --- | --- | --- | --- | --- | --- | --- | --- | --- | --- | --- | --- | --- | --- | --- | --- | --- | --- | --- | --- | --- | --- | --- | --- | --- |

1. R for resistant, S for susceptible; using CLSI guidelines*^6^*
