# Supplementary material for: Low-Level Antimicrobials in the Medicinal Leech Select for Resistant Pathogens That Spread to Patients
Source: mBio. 2018 Jul 24;9(4):e01328-18. doi: 10.1128/mBio.01328-18 (PMC6058295; doi:10.1128/mBio.01328-18)
Supplement: TABLE S2 [file mbo004183985st2.docx]

**Supplementary Table 2. Published case reports of Cp^R^ *Aeromonas* spp. cultured in association with medicinal leech therapy.**

| \| Reference \| No. of case reports \| Patient Case Conditions \| Prophylaxis Used \| Treatment that cleared infection \| Isolate(s) Cultured \| Geographic Location \| Year Published \| \| --- \| --- \| --- \| --- \| --- \| --- \| --- \| --- \| \|  \|  \|  \|  \|  \|  \|  \|  \| \| Wang *et al.,* 2011 \| 1 \| Mandibulectomy with planned tissue flap reconstruction, infection and necrosis of the flap \| ciprofloxacin \| cefepime \| *A. hydrophila* \| Missouri, USA \| 2011 \| \|  \|  \|  \|  \|  \|  \|  \|  \| \| Sartor *et al.,* 2013 \| 1 \| Infection of skin flap of hand crush injury \| ciprofloxacin \| cotrimoxazole \| *A. hydrophila* \| Marseille, France \| 2013 \| \| Giltner *et al.,* 2013 \| 1 \| Mandibular osteotomy, necrosis of mandibular flap and wound surrounding the distraction arm device \| ciprofloxacin \| vancomycin \| *A. hydrophila, Morganella morganii* \| California, USA \| 2013 \| \|  \|  \|  \|  \|  \|  \|  \|  \| \| Wilmer *et al.,* 2013 \| 1 \| Amputation of three digits and necrosis of amputation sites \| ciprofloxacin \| co-trimoxazole \| *A. hydrophila* \| British Columbia, Canada \| 2013 \| \|  \|  \|  \|  \|  \|  \|  \|  \| \| Patel *et al.,* 2013 \| 1 \| Breast reconstruction, infection of the implant \| ciprofloxacin, vancomycin \| aztreonam \| *A. hydrophila* \| Washington D.C., USA \| 2013 \| \| van Alphen *et al.*, 2014 \| 2 \| Replantation of 4 fingers resulting flap necrosis after leech therapy, followed by amputation \| ertapenem \| ceftriaxone and co-trimoxazole \| *A. hydrophila* \| Minnesota, USA \| 2014 \| \| Replantation of 2 fingers failed following leech therapy, amputation \| ciprofloxacin \| cefepime, metronidazole, vancomycin; followed by ceftriaxone \| *A. hydrophila, Proteus vulgaris, Morganella morganii* \| \| Ruppé *et al.,* 2018 \| 1 \| Replantation of thumb procedure followed by leech therapy for venous congestion, complicated by *Aeromonas* infection \| piperacillin/tazobactam  and later, cotrimoxazole \| N/A but isolate was resistant to many antibiotics, including ciprofloxacin \| *A. salmonicida* \| Geneva, Switzerland \| 2018 \| \|  \|  \|  \|  \|  \|  \|  \|  \| |
| --- | --- | --- | --- | --- | --- | --- | --- | --- | --- | --- | --- | --- | --- | --- | --- | --- | --- | --- | --- | --- | --- | --- | --- | --- | --- | --- | --- | --- | --- | --- | --- | --- | --- | --- | --- | --- | --- | --- | --- | --- | --- | --- | --- | --- | --- | --- | --- | --- | --- | --- | --- | --- | --- | --- | --- | --- | --- | --- | --- | --- | --- | --- | --- | --- | --- | --- | --- | --- | --- | --- | --- | --- | --- | --- | --- | --- | --- | --- | --- | --- | --- | --- | --- | --- | --- | --- | --- | --- | --- | --- | --- | --- | --- | --- | --- | --- | --- | --- | --- | --- | --- | --- | --- | --- | --- | --- | --- | --- |
