# Supplementary material for: Low-Level Antimicrobials in the Medicinal Leech Select for Resistant Pathogens That Spread to Patients
Source: mBio. 2018 Jul 24;9(4):e01328-18. doi: 10.1128/mBio.01328-18 (PMC6058295; doi:10.1128/mBio.01328-18)
Supplement: TABLE S3 [file mbo004183985st3.docx]

**Supplementary Table 3. Description of isolate sources.** Origin of clinical isolates or medicinal leech shipments from which leech-derived strains have been isolated and sequenced using Illumina MiSeq (or PacBio in the case of Hv13-B-10d), and identified using HKG and ANI-based methods. Cp MIC refers to the minimum inhibitory concentration of each strain to ciprofloxacin (µg/ml). Resistance levels R, I, and S mean resistant to Cp, intermediately resistant and susceptible, respectively.

| Strain | Cp MIC (μg/ml) | Resistance Level | Source*^*^* | Reference |
| --- | --- | --- | --- | --- |
| Clinical |  |  |  |  |
| *A. hydrophila* |  |  |  |  |
| CA-13-1 | ≥32 | R | Wound | Giltner et al., 2013 |
| CA-13-2 | 8 | R | Surgical instrument | Giltner et al., 2013 |
| IA-13-1 | 4 | R | Wound | This study |
| IA-13-2 | ≥32 | R | Wound | This study |
| LR-12-1 | ≥32 | R | Wound | This study |
|  |  |  |  |  |
| LR-12-2 | 1 | S | Aquarium | This study |
| MO-11-1 | ≥32 | R | Wound | Wang et al., 2011 |
| *A. veronii* |  |  |  |  |
| LR-14-3 | 8 | R | Wound | This study |
| LR-14-4 | 8 | R | Wound | This study |
| *Aeromonas* sp. |  |  |  |  |
| CA-13-4 | 0.004 | S | Aquarium | Giltner et al., 2013 |
| Leech-Derived |  |  |  |  |
| *A. hydrophila* |  |  |  |  |
| Hv12-A-03a | 8 | R | Distributor | This study |
| Hv13-B-10d | ≥32 | R | Distributor | This study |
| *A. veronii* |  |  |  |  |
| Hv13-B-08a | 16 | R | Distributor | This study |
| Hv13-B-10a | 0.5 | S | Distributor | This study |
| Hv13-B-10c | 16 | R | Distributor | This study |
| Hv13-B-11a | 1 | S | Distributor | This study |
| Hv13-B-13a | 16 | R | Distributor | This study |
| Hv13-B-13b | ≥32 | R | Distributor | This study |
| Hv13-C-09a | 2 | I | Distributor | This study |
| Hv13-C-10a | 16 | R | Distributor | This study |
| Hv13-C-10b | 4 | R | Distributor | This study |
| Hv13-D-07a | 2 | I | Distributor | This study |
| Hv13-E-01a | ≥32 | R | Farm | This study |
| Hv13-E-04a | 8 | R | Farm | This study |
| Hv13-E-06a | ≥32 | R | Farm | This study |
| Hv13-F-06a | 0.004 | S | Farm | This study |
| Hv14-G-10a | 4 | R | Distributor | This study |
| Hv15-H-03a | 8 | R | Farm | This study |
| Hv15-I-03a | 1 | S | Farm | This study |
| Hv15-J-01a | 0.25 | S | Farm | This study |
| Hv15-J-02a | ≥32 | R | Farm | This study |
| Hv15-J-03a | 4 | R | Farm | This study |
| Leech Control Isolates |  |  |  |  |
| Hm21 | 0.008 | S | Farm | Graf, 1999 |
| Hm22 | 0.004 | S | Farm | Graf, 1999 |
| Hm225 | 0.002 | S | Farm | Graf, 1999 |
| Hm231 | 0.004 | S | Farm | Graf, 1999 |
| Hm241 | 0.002 | S | Farm | Graf, 1999 |
| Hm571 | 0.002 | S | Farm | Graf, 1999 |
| G3-C1 | 0.002 | S | Another farm in Europe | This study |

* For clinical strains, source refers to the site from which strain was isolated (patient wound, surgical device, or aquarium in which medicinal leech was housed). For leech-derived strains, source describes where the leech shipment originated (the main FDA-approved distributor or leech-raising farm which supplies the distributor).
