# Supplementary material for: Low-Level Antimicrobials in the Medicinal Leech Select for Resistant Pathogens That Spread to Patients
Source: mBio. 2018 Jul 24;9(4):e01328-18. doi: 10.1128/mBio.01328-18 (PMC6058295; doi:10.1128/mBio.01328-18)
Supplement: TABLE S4 [file mbo004183985st4.docx]

**Supplementary Table 4. Detection of fluoroquinolones in leech gut content.** Ciprofloxacin (Cp) and enrofloxacin (Ef) in leech crop content of a 2014 shipment from the FDA-approved distributor were quantified using HPLC/MS/MS. The limit of detection ranged between 0.002–0.0012 µg/mL for 100 µL of ILF and 0.004–0.024 μg/mL for 50μL*. The relative standard deviation was determined using error propagation from the curve fit and technical replicates.

| \| Leech \| Cp μg/mL \|  \| Relative standard deviation μg/mL \| Ef μg/mL \|  \| Relative standard deviation μg/mL \| \| --- \| --- \| --- \| --- \| --- \| --- \| --- \| \|  \|  \|  \|  \|  \|  \|  \| \| 1 \| 0.0178 \| ± \| 0.0005 \| 0.00976 \| ± \| 0.0004 \| \| 2* \| 0.0202 \| ± \| 0.0003 \| 0.02664 \| ± \| 0.0005 \| \| 3 \| 0.0184 \| ± \| 0.0005 \| 0.00798 \| ± \| 0.0004 \| \| 4 \| 0.0178 \| ± \| 0.0005 \| 0.00648 \| ± \| 0.0003 \| \| 5* \| 0.0203 \| ± \| 0.0003 \| 0.02352 \| ± \| 0.0005 \| \| 6 \| 0.0132 \| ± \| 0.0004 \| 0.01078 \| ± \| 0.0004 \| \| 7 \| 0.0212 \| ± \| 0.0006 \| 0.00528 \| ± \| 0.0003 \| \| 8 \| 0.0156 \| ± \| 0.0005 \| 0.00682 \| ± \| 0.0003 \| \| 9 \| 0.0166 \| ± \| 0.0005 \| 0.01898 \| ± \| 0.0007 \| \| 10 \| 0.0404 \| ± \| 0.0011 \| 0.01478 \| ± \| 0.0006 \| |
| --- | --- | --- | --- | --- | --- | --- | --- | --- | --- | --- | --- | --- | --- | --- | --- | --- | --- | --- | --- | --- | --- | --- | --- | --- | --- | --- | --- | --- | --- | --- | --- | --- | --- | --- | --- | --- | --- | --- | --- | --- | --- | --- | --- | --- | --- | --- | --- | --- | --- | --- | --- | --- | --- | --- | --- | --- | --- | --- | --- | --- | --- | --- | --- | --- | --- | --- | --- | --- | --- | --- | --- | --- | --- | --- | --- | --- | --- | --- | --- | --- | --- | --- | --- | --- |
