# Supplementary material for: Low-Level Antimicrobials in the Medicinal Leech Select for Resistant Pathogens That Spread to Patients
Source: mBio. 2018 Jul 24;9(4):e01328-18. doi: 10.1128/mBio.01328-18 (PMC6058295; doi:10.1128/mBio.01328-18)

**Supplementary Figure 1. Average Nucleotide Identity (ANI) and MLSA distance values.**

The lower triangle displays ANI values and the upper triangle displays uncorrected number of differences in the alignment used to create the MLSA phylogeny. ANI values are deep red (>= 99% ANI), red (>=98% ANI), and orange (>=96% ANI). Difference colors are dark green (0 differences), medium green (<3 differences), and light green (< 20 differences).


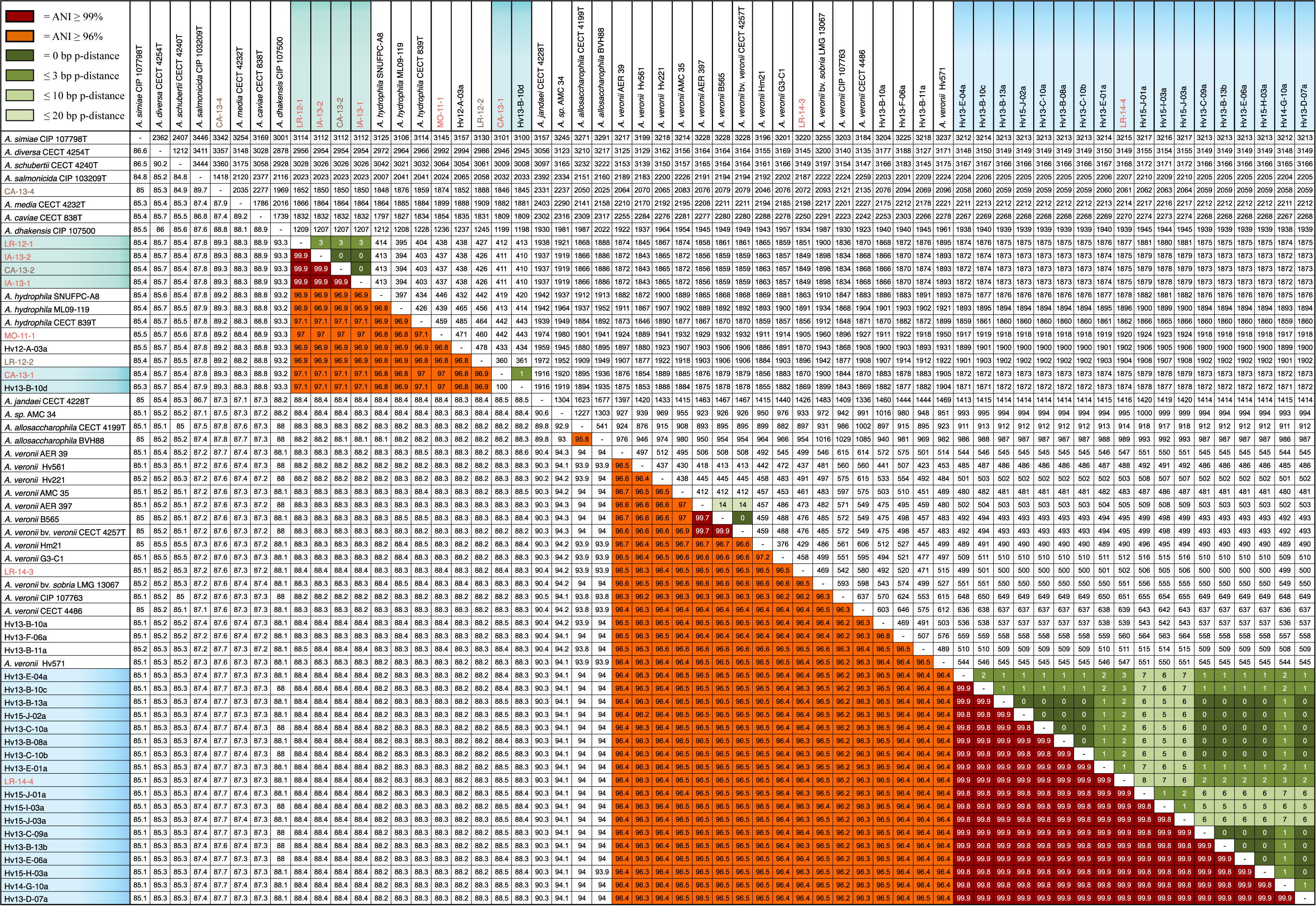

Supplement: FIG S1 [file mbo004183985sf1.docx]
