# Supplementary material for: Low-Level Antimicrobials in the Medicinal Leech Select for Resistant Pathogens That Spread to Patients
Source: mBio. 2018 Jul 24;9(4):e01328-18. doi: 10.1128/mBio.01328-18 (PMC6058295; doi:10.1128/mBio.01328-18)
Supplement: TABLE S5 [file mbo004183985st5.docx]

**Supplementary Table 5. Description of clinical and leech isolates sequenced in this study.** (continued on next page)

| \| **Strain** \| **Species** \| **Cp^R^** \| **Leech no.** \| **Ship-ment date** \| **Avg genome coverage *^d^*** \| **No. of scaffolds** \| **Genome size (Mbp)** \| **N50*^e^* (nt)** \| **Biosample Accession ID** \| **BioProject _**  **ID** \|  \| \| --- \| --- \| --- \| --- \| --- \| --- \| --- \| --- \| --- \| --- \| --- \| --- \| \| Clinical \|  \|  \|  \|  \|  \|  \|  \|  \|  \|  \|  \| \| CA-13-1 \| *A. hydrophila* \| R \| N/A \| N/A \| 57 \| 70 \| 4.74 \| 157,731 \| SAMN03742454 \| PRJNA297179 \|  \| \| CA-13-2 \| *A. hydrophila* \| R \| N/A \| N/A \| 59 \| 120 \| 4.8 \| 91,036 \| SAMN04123813 \| PRJNA297409 \|  \| \| CA-13-4 \| *Aeromonas ^c^* \| S \| N/A \| N/A \| 90 \| 41 \| 4.87 \| 258,555 \| N/A \| PRJEB6940 \|  \| \| IA-13-1 \| *A. hydrophila* \| R \| N/A \| N/A \| 82 \| 68 \| 4.81 \| 151,183 \| SAMN04123814 \| PRJNA297409 \|  \| \| IA-13-2 \| *A. hydrophila* \| R \| N/A \| N/A \| 30 \| 153 \| 4.83 \| 72,900 \| SAMN04123815 \| PRJNA297409 \|  \| \| LR-12-1 \| *A. hydrophila* \| R \| N/A \| N/A \| 96 \| 150 \| 4.83 \| 91,054 \| SAMN04123816 \| PRJNA297409 \|  \| \| LR-12-2 \| *A. hydrophila* \| S \| N/A \| N/A \| 70 \| 102 \| 4.8 \| 129,173 \| SAMN04123817 \| PRJNA297409 \|  \| \| LR-14-3 \| *A. veronii* \| R \| N/A \| N/A \| 76 \| 58 \| 4.7 \| 164,376 \| SAMN04123818 \| PRJNA297409 \|  \| \| LR-14-4 \| *A. veronii* \| R \| N/A \| N/A \| 98 \| 131 \| 4.95 \| 130,291 \| SAMN04123819 \| PRJNA297409 \|  \| \| MO-11-1 *^a^* \| *A. hydrophila* \| R \| N/A \| N/A \| 59 \| 74 \| 5.02 \| 128,277 \| SAMN04123820 \| PRJNA297409 \|  \| \| Leech \|  \|  \|  \|  \|  \|  \|  \|  \|  \|  \|  \| \| Hv12-A-03a \| *A. hydrophila* \| R \| A3 \| Dec-12 \| 224 \| 119 \| 4.74 \| 98,866 \| SAMN04123821 \| PRJNA297409 \|  \| \| Hv13-B-08a \| *A. veronii* \| R \| B8 \| Feb-13 \| 198 \| 126 \| 4.94 \| 113,140 \| SAMN04123823 \| PRJNA297409 \|  \| \| Hv13-B-10a \| *A. veronii* \| S \| B10 \| Feb-13 \| 72 \| 157 \| 4.8 \| 62,521 \| SAMN04123824 \| PRJNA297409 \|  \| \| Hv13-B-10c \| *A. veronii* \| R \| B10 \| Feb-13 \| 131 \| 173 \| 4.94 \| 69,154 \| SAMN04123825 \| PRJNA297409 \|  \| \| Hv13-B-10d *^b^* \| *A. hydrophila* \| R \| B10 \| Feb-13 \| N/A \| N/A *^b^* \| 4.79 \| N/A *^b^* \| SAMN04123822 \| PRJNA297409 \|  \| \| Hv13-B-11a \| *A. veronii* \| S \| B11 \| Feb-13 \| 140 \| 87 \| 4.96 \| 164,556 \| SAMN04123826 \| PRJNA297409 \|  \| \| Hv13-B-13a \| *A. veronii* \| R \| B13 \| Feb-13 \| 102 \| 142 \| 4.95 \| 101,118 \| SAMN04123827 \| PRJNA297409 \|  \| \| Hv13-B-13b \| *A. veronii* \| R \| B13 \| Feb-13 \| 194 \| 127 \| 4.94 \| 120,574 \| SAMN04123828 \| PRJNA297409 \|  \| \| Hv13-C-09a \| *A. veronii* \| I \| C9 \| Mar-13 \| 117 \| 132 \| 4.9 \| 105,981 \| SAMN04123829 \| PRJNA297409 \|  \| \| Hv13-C-10a \| *A. veronii* \| R \| C10 \| Mar-13 \| 278 \| 119 \| 4.95 \| 136,584 \| SAMN04123830 \| PRJNA297409 \|  \| \| Hv13-C-10b \| *A. veronii* \| R \| C10 \| Mar-13 \| 255 \| 124 \| 4.95 \| 122,328 \| SAMN04123831 \| PRJNA297409 \|  \| \| Hv13-D-07a \| *A. veronii* \| I \| D7 \| Jun-13 \| 118 \| 136 \| 4.94 \| 130,668 \| SAMN04123832 \| PRJNA297409 \|  \| \| Hv13-E-01a \| *A. veronii* \| R \| E1 \| Jun-13 \| 101 \| 143 \| 4.93 \| 108,313 \| SAMN04123833 \| PRJNA297409 \|  \| \| Hv13-E-04a \| *A. veronii* \| R \| E4 \| Jun-13 \| 113 \| 122 \| 4.93 \| 136,420 \| SAMN04123834 \| PRJNA297409 \|  \| \| Hv13-E-06a \| *A. veronii* \| R \| E6 \| Jun-13 \| 154 \| 133 \| 4.94 \| 105,984 \| SAMN04123835 \| PRJNA297409 \|  \| \| Hv13-F-06a \| *A. veronii* \| S \| F6 \| Feb-13 \| 76 \| 81 \| 4.68 \| 111,930 \| SAMN04123836 \| PRJNA297409 \|  \| \| Hv14-G-10a \| *A. veronii* \| I \| G10 \| Nov-14 \| 45 \| 177 \| 4.94 \| 68,684 \| SAMN04123837 \| PRJNA297409 \|  \| \| Hv15-H-03a \| *A. veronii* \| R \| H3 \| Dec-14 \| 85 \| 142 \| 4.95 \| 101,427 \| SAMN04123838 \| PRJNA297409 \|  \| \| Hv15-I-03a \| *A. veronii* \| S \| I3 \| Dec-14 \| 185 \| 124 \| 4.97 \| 120,636 \| SAMN04123839 \| PRJNA297409 \|  \| \| Hv15-J-01a \| *A. veronii* \| S \| J1 \| Dec-14 \| 85 \| 148 \| 4.96 \| 71,974 \| SAMN04123840 \| PRJNA297409 \|  \| \| Hv15-J-02a \| *A. veronii* \| R \| J2 \| Dec-14 \| 270 \| 144 \| 4.97 \| 99,797 \| SAMN04123841 \| PRJNA297409 \|  \| \| Hv15-J-03a \| *A. veronii* \| R \| J3 \| Dec-14 \| 95 \| 153 \| 4.98 \| 83,387 \| SAMN04123842 \| PRJNA297409 \|  \| \| Hm561 \| *A. veronii* \| S \| N/A \| N/A \| 240 \| 62 \| 5 \| 305,599 \| SAMN04123844 \| PRJNA297409 \|  \| \| Hm571 \| *A. veronii* \| S \| N/A \| N/A \| 47 \| 62 \| 4.93 \| 174,508 \| SAMN04123845 \| PRJNA297409 \|  \| \| G3-C1 \| *A. veronii* \| S \| N/A \| N/A \| 64 \| 56 \| 4.76 \| 161,764 \| SAMN04123846 \| PRJNA297409 \|  \| \| Hm221 \| *A. veronii* \| S \| N/A \| N/A \| 189 \| 54 \| 4.86 \| 206,668 \| SAMN04123843 \| PRJNA297409 \|  \| \| Hm21*^f^* \| *A. veronii* \| S \| N/A \| N/A \| 200 \| 50 \| 4.68 \| 179,631 \| ATFB01 \| N/A \|  \| |
| --- | --- | --- | --- | --- | --- | --- | --- | --- | --- | --- | --- | --- | --- | --- | --- | --- | --- | --- | --- | --- | --- | --- | --- | --- | --- | --- | --- | --- | --- | --- | --- | --- | --- | --- | --- | --- | --- | --- | --- | --- | --- | --- | --- | --- | --- | --- | --- | --- | --- | --- | --- | --- | --- | --- | --- | --- | --- | --- | --- | --- | --- | --- | --- | --- | --- | --- | --- | --- | --- | --- | --- | --- | --- | --- | --- | --- | --- | --- | --- | --- | --- | --- | --- | --- | --- | --- | --- | --- | --- | --- | --- | --- | --- | --- | --- | --- | --- | --- | --- | --- | --- | --- | --- | --- | --- | --- | --- | --- | --- | --- | --- | --- | --- | --- | --- | --- | --- | --- | --- | --- | --- | --- | --- | --- | --- | --- | --- | --- | --- | --- | --- | --- | --- | --- | --- | --- | --- | --- | --- | --- | --- | --- | --- | --- | --- | --- | --- | --- | --- | --- | --- | --- | --- | --- | --- | --- | --- | --- | --- | --- | --- | --- | --- | --- | --- | --- | --- | --- | --- | --- | --- | --- | --- | --- | --- | --- | --- | --- | --- | --- | --- | --- | --- | --- | --- | --- | --- | --- | --- | --- | --- | --- | --- | --- | --- | --- | --- | --- | --- | --- | --- | --- | --- | --- | --- | --- | --- | --- | --- | --- | --- | --- | --- | --- | --- | --- | --- | --- | --- | --- | --- | --- | --- | --- | --- | --- | --- | --- | --- | --- | --- | --- | --- | --- | --- | --- | --- | --- | --- | --- | --- | --- | --- | --- | --- | --- | --- | --- | --- | --- | --- | --- | --- | --- | --- | --- | --- | --- | --- | --- | --- | --- | --- | --- | --- | --- | --- | --- | --- | --- | --- | --- | --- | --- | --- | --- | --- | --- | --- | --- | --- | --- | --- | --- | --- | --- | --- | --- | --- | --- | --- | --- | --- | --- | --- | --- | --- | --- | --- | --- | --- | --- | --- | --- | --- | --- | --- | --- | --- | --- | --- | --- | --- | --- | --- | --- | --- | --- | --- | --- | --- | --- | --- | --- | --- | --- | --- | --- | --- | --- | --- | --- | --- | --- | --- | --- | --- | --- | --- | --- | --- | --- | --- | --- | --- | --- | --- | --- | --- | --- | --- | --- | --- | --- | --- | --- | --- | --- | --- | --- | --- | --- | --- | --- | --- | --- | --- | --- | --- | --- | --- | --- | --- | --- | --- | --- | --- | --- | --- | --- | --- | --- | --- | --- | --- | --- | --- | --- | --- | --- | --- | --- | --- | --- | --- | --- | --- | --- | --- | --- | --- | --- | --- | --- | --- | --- | --- | --- | --- | --- | --- | --- | --- | --- | --- | --- | --- | --- | --- | --- | --- | --- | --- | --- | --- | --- | --- | --- | --- | --- | --- | --- | --- | --- | --- | --- | --- | --- | --- | --- | --- | --- | --- | --- | --- | --- | --- | --- | --- | --- | --- | --- | --- | --- | --- | --- | --- | --- | --- | --- | --- | --- | --- | --- | --- | --- | --- | --- | --- | --- | --- | --- | --- | --- | --- | --- | --- | --- | --- | --- |

1. Sequenced by the Human Microbiome U54 initiative, Broad Institute (broadinstitute.org) using Illumina; Genbank BioProject 71511.
2. Strains were sequenced using an Illumina MiSeq and assembled with CLC Genomics Workbench except for strain Hv13-B-10d, which is a closed genome sequenced with PacBio and assembled using Celera.
3. Likely new Aeromonas sp. see Colston et al., 2014.
4. Total bp sequenced divided by total genome size.
5. Shortest contig length (nucleotides) of the longest contigs covering 50% total contigs-size.
6. Hm21 previously registered in Genbank, National Center for Biotechnology Information Accession no. ATFB01; See reference Bomar et al., 2013 [20]
