# Supplementary material for: Low-Level Antimicrobials in the Medicinal Leech Select for Resistant Pathogens That Spread to Patients
Source: mBio. 2018 Jul 24;9(4):e01328-18. doi: 10.1128/mBio.01328-18 (PMC6058295; doi:10.1128/mBio.01328-18)
Supplement: TABLE S6 [file mbo004183985st6.docx]

**Supplementary Table 6. API 20 NE strip phenotypic testing results shown below for *Aeromonas* isolates sequenced in this study**^a^**.** (continued on next page)

| Strain | Significant Taxon | T value ^b^ | % ID ^b^ | NO2 | TRP | GLU | ADH | URE | ESC | GEL | PNG | GLU | ARA | MNE | MAN | NAG | MAL | GNT | CAP | ADI | MLT | CIT | PAC | OX |
| --- | --- | --- | --- | --- | --- | --- | --- | --- | --- | --- | --- | --- | --- | --- | --- | --- | --- | --- | --- | --- | --- | --- | --- | --- |
| *Clinical Isolates* | |  |  |  |  |  |  |  |  |  |  |  |  |  |  |  |  |  |  |  |  |  |  |  |
| MO-11-1 | *A. sobria* | 0.72 | 84.80% | + | + | + | + | - | + | + | + | + | + | + | + | + | + | + | + | - | + | - | - | + |
| LR-12-1 | *A. hydrophila* | 1 | 99.90% | + | + | + | + | - | + | + | + | + | + | + | + | + | + | + | + | - | + | - | - | + |
| LR-12-2 | *A. hydrophila* | 1 | 99.90% | + | + | + | + | - | + | + | + | + | + | + | + | + | + | + | + | - | + | - | - | + |
| CA-13-1 | *A. hydrophila* | 1 | 99.90% | + | + | + | + | - | + | + | + | + | + | + | + | + | + | + | + | - | + | - | - | + |
| CA-13-2 | *A. hydrophila* | 1 | 99.90% | + | + | + | + | - | + | + | + | + | + | + | + | + | + | + | + | - | + | - | - | + |
| CA-13-4 ^c^ | *A. hydrophila/caviae* | 0.63 | 99.80% | + | + | + | + | - | + | + | - | + | + | + | + | + | + | + | - | - | + | - | - | + |
| IA-13-1 | *A. hydrophila* | 1 | 99.90% | + | + | + | + | - | + | + | + | + | + | + | + | + | + | + | + | - | + | - | - | + |
| IA-13-2 | *A. hydrophila* | 1 | 99.90% | + | + | + | + | - | + | + | + | + | + | + | + | + | + | + | + | - | + | - | - | + |
| LR-14-3 | *A. sobria* | 0.87 | 99.40% | + | + | + | + | - | - | + | + | + | - | + | + | + | + | + | + | - | + | + | - | + |
| LR-14-4 | *A. sobria* | 0.35 | 65.60% | + | + | - | - | - | - | + | + | + | - | + | + | + | + | + | - | - | + | + | - | + |
| *Leech-derived Isolates* | |  |  |  |  |  |  |  |  |  |  |  |  |  |  |  |  |  |  |  |  |  |  |  |
| Hv12-A-03a | *A. hydrophila* | 1 | 99.90% | + | + | + | + | - | + | + | + | + | + | + | + | + | + | + | + | - | + | - | - | + |
| Hv13-B-10d | *A. hydrophila* | 1 | 99.90% | + | + | + | + | + | + | + | + | + | + | + | + | + | + | + | + | - | + | - | - | + |
| Hv13-C-10a | *A. sobria* | 0.72 | 84.70% | + | + | + | + | - | - | + | + | + | - | + | + | + | + | + | - | - | + | - | - | + |
| Hv13-C-10b | *A. sobria* | 0.72 | 84.80% | + | + | + | + | - | - | + | + | + | - | + | + | + | + | + | - | - | + | - | - | + |
| Hv13-E-04a | *A. hydrophila* | 0.46 | 99.50% | + | + | + | + | + | + | + | + | + | - | + | + | + | + | + | - | - | + | - | - | + |
| Hv13-E-06a | *V. parahaemolyticus* | 0.56 | 61.20% | + | + | - | - | - | - | + | + | + | - | + | + | + | + | + | - | - | + | - | - | + |
| Hv13-D-07a | *A. sobria* | 0.5 | 94.30% | + | + | - | + | - | - | + | + | + | - | + | + | + | + | + | - | - | + | - | - | + |
| Hv13-B-11a | *A. sobria* | 0.82 | 97.50% | + | + | + | + | - | - | + | + | + | - | + | + | + | + | + | - | - | + | + | - | + |
| Hv13-B-13a | *A. sobria* | 0.72 | 84.80% | + | + | + | + | - | - | + | + | + | - | + | + | + | + | + | - | - | + | - | - | + |
| Hv13-B-13b | *V. parahaemolyticus* | 0.9 | 83.80% | + | + | + | - | - | - | + | + | + | - | + | + | + | + | + | - | - | + | - | - | + |
| Hv13-E-01a | *A. hydrophila/caviae* | 0.18 | 99.50% | + | + | - | + | + | + | + | + | + | - | + | + | + | + | + | - | - | + | - | - | + |
| Hv13-B-08a | *A. hydrophila/caviae* | 0.74 | 58.20% | + | + | + | + | - | - | + | + | + | + | + | + | + | + | + | - | - | + | - | - | + |
| Hv13-B-10c | *A. sobria* | 0.61 | 99.10% | + | + | - | + | - | - | + | + | + | - | + | + | + | + | + | - | - | + | + | - | + |
| Hv13-B-10a | *A. hydrophila/caviae* | 0.74 | 58.20% | + | + | + | + | - | - | + | + | + | + | + | + | + | + | + | - | - | + | - | - | + |
| Hv13-F-06a | *A. sobria* | 0.9 | 94.90% | + | + | + | + | - | - | + | + | + | - | + | + | + | + | + | + | - | + | - | - | + |
| Hv15-J-02a | *A. sobria* | 0.59 | 90.40% | + | + | + | + | - | - | + | + | + | - | + | + | + | + | + | - | - | + | - | - | + |
| Hv15-J-03a | *A. sobria* | 0.25 | 42.10% | + | + | - | - | - | - | + | + | + | - | + | + | + | + | + | - | - | + | - | - | + |
| Hv15-H-03a | *A. sobria* | 0.26 | 59.20% | + | + | + | + | - | - | + | + | + | - | + | + | + | + | - | - | - | + | - | - | + |
| Hv15-I-03a | *A. sobria* | 0.69 | 98.50% | + | + | + | + | - | - | + | + | + | - | + | + | + | + | + | - | - | + | + | - | + |
| Hv15-J-01a | *A. sobria* | 0.82 | 97.50% | + | + | + | + | - | - | + | + | + | - | + | + | + | + | + | - | - | + | + | - | + |
| Hv14-G-10a | *A. sobria* | 0.82 | 97.50% | + | + | + | + | - | - | + | + | + | - | + | + | + | + | + | - | - | + | + | - | + |
| Hv13-C-09a | *A. sobria* | 0.72 | 84.70% | + | + | + | + | - | - | + | + | + | - | + | + | + | + | + | - | - | + | - | - | + |
| *Leech Control Isolates* | |  |  |  |  |  |  |  |  |  |  |  |  |  |  |  |  |  |  |  |  |  |  |  |
| Hm21 | *A. sobria* | 0.82 | 97.50% | + | + | + | + | - | - | + | + | + | - | + | + | + | + | + | - | - | + | + | - | + |
| Hm571 | *A. hydrophila/caviae* | 0.86 | 83.90% | + | + | + | + | - | + | + | + | + | - | + | + | + | + | + | + | - | + | + | - | + |
| Hm561 | *A. sobria* | 0.5 | 92.00% | + | + | + | + | + | - | + | + | + | - | + | + | + | + | + | + | - | + | + | - | + |
| G3-C1 | *A. sobria* | 1 | 99.20% | + | + | + | + | - | - | + | + | + | - | + | + | + | + | + | + | - | + | + | - | + |
| Hm221 | *A. sobria* | 1 | 99.20% | + | + | + | + | - | - | + | + | + | - | + | + | + | + | + | + | - | + | + | - | + |

1. **Reduction and enzymatic activity tests:** NO_2_= nitrate; TRP=L-tryptophane; GLU= D-glucose; ADH=L-arginine; URE=urea; ESC= esculin ferric citrate; GEL= gelatin; PNPG= 4-nitrophenyl-βD-galactopyranoside.

**Assimilation/fermentation tests:** GLU= D-glucose; ARA= L-arabinose; MNE= D-mannose; MAN= D-mannitol; NAG= N-acetyl-glucosamine; MAL= D-maltose; GNT= potassium gluconate; CAP= capric acid; ADI= adipic acid; MLT= malic acid; CIT= trisodium citrate; PAC= phenylacetic acid. **OX**= oxidase test.

1. According to the manufacturer's instructions, strain identification is measured using the following criteria: excellent species identification, percent identification of ≥99.9% and *T* value of ≥0.75; very good species identification, percent identification of ≥99.0% and *T* value of ≥0.5; and good species identification, percent identification of ≥90.0% and *T* value of ≥0.25; acceptable species identification, percent identification of ≥80.0% and *T* value of ≥0.0
2. Previously published in Colston et al., 2014
