# Supplementary material for: Low-Level Antimicrobials in the Medicinal Leech Select for Resistant Pathogens That Spread to Patients
Source: mBio. 2018 Jul 24;9(4):e01328-18. doi: 10.1128/mBio.01328-18 (PMC6058295; doi:10.1128/mBio.01328-18)
Supplement: TABLE S7 [file mbo004183985st7.docx]

**Supplementary Table 7. MICs of ciprofloxacin for all *Aeromonas* isolates included in Fig. 1 determined by E-tests.** (continued on next page)

| \| Species \| Strain \| Cp MIC (μg/mL) ^a^ \| \| --- \| --- \| --- \| \| Clinical \|  \|  \| \| *A. hydrophila* \| CA-13-1 \| ≥32 \| \| *A. hydrophila* \| CA-13-2 \| 8 \| \| *A. hydrophila* \| IA-13-1 \| 4 \| \| *A. hydrophila* \| IA-13-2 \| ≥32 \| \| *A. hydrophila* \| LR-12-2 \| 1 \| \| *A. hydrophila* \| MO-11-1 \| ≥32 \| \| *A. hydrophila* \| LR-12-1 \| ≥32 \| \| *A. veronii* \| LR-14-3 \| 8 \| \| *A. veronii* \| LR-14-4 \| 8 \| \| *Aeromonas sp.* \| CA-13-4 \| 0.004 \| \| Other isolates \|  \|  \| \| *A. allosaccharophila* \| BVH88 \| 0.002 \| \| *A. allosaccharophila* \| CECT4199 \| 0.004 \| \| *A. hydrophila* \| ATCC7966 \| 0.004 \| \| *A. hydrophila* \| CIP1075 \| 0.008 \| \| *A. hydrophila* \| Hv14-G-10a \| 4 \| \| *A. hydrophila* \| Hv12-A-03a \| 8 \| \| *A. hydrophila* \| Hv13-B-10d \| ≥32 \| \| *A. jandaei* \| F314 \| 0.002 \| \| *A. veronii* \| Hm571 \| 0.002 \| \| *A. veronii* \| Hm561 \| 0.002 \| \| *A. veronii* \| G3-C1 \| 0.002 \| \| *A. veronii* \| AER397 \| 0.002 \| \| *A. veronii* \| CIP107763 \| 0.002 \| \| *A. veronii* \| AMC34 \| 0.004 \| \| *A. veronii* \| CECT4486 \| 0.004 \| \| *A. veronii* \| AMC35 \| 0.004 \| \| A. veronii \| Hv13-F-06a \| 0.004 \| \| *A. veronii* \| AER39 \| 0.004 \| \| *A. veronii* \| Hm221 \| 0.008 \| \| A. veronii \| Hm21 \| 0.008 \| \| *A. veronii* \| LMG13067 \| 0.064 \| \| *A. veronii* \| Hv15-J-01a \| 0.25 \| \| *A. veronii* \| Hv13-B-10a \| 0.50 \| \| *A. veronii* \| Hv15-I-03a \| 1 \| \| *A. veronii* \| Hv13-B-11a \| 1 \| \| *A. veronii* \| Hv13-C-09a \| 2 \| \| *A. veronii* \| Hv13-D-07a \| 2 \| \| *A. veronii* \| Hv15-J-03a \| 4 \| \| *A. veronii* \| Hv13-C-10b \| 4 \| \| *A. veronii* \| Hv15-H-03a \| 8 \| \| *A. veronii* \| Hv13-E-04a \| 8 \| \| *A. veronii* \| Hv13-B-10c \| 16 \| \| *A. veronii* \| Hv13-C-10a \| 16 \| \| *A. veronii* \| Hv13-B-08a \| 16 \| \| *A. veronii* \| Hv13-B-13a \| 16 \| \| *A. veronii* \| Hv15-J-02a \| ≥32 \| \| *A. veronii* \| Hv13-B-13b \| ≥32 \| \| *A. veronii* \| Hv13-E-01a \| ≥32 \| \| *A. veronii* \| Hv13-E-06a \| ≥32 \| \|  \|  \|  \| |
| --- | --- | --- | --- | --- | --- | --- | --- | --- | --- | --- | --- | --- | --- | --- | --- | --- | --- | --- | --- | --- | --- | --- | --- | --- | --- | --- | --- | --- | --- | --- | --- | --- | --- | --- | --- | --- | --- | --- | --- | --- | --- | --- | --- | --- | --- | --- | --- | --- | --- | --- | --- | --- | --- | --- | --- | --- | --- | --- | --- | --- | --- | --- | --- | --- | --- | --- | --- | --- | --- | --- | --- | --- | --- | --- | --- | --- | --- | --- | --- | --- | --- | --- | --- | --- | --- | --- | --- | --- | --- | --- | --- | --- | --- | --- | --- | --- | --- | --- | --- | --- | --- | --- | --- | --- | --- | --- | --- | --- | --- | --- | --- | --- | --- | --- | --- | --- | --- | --- | --- | --- | --- | --- | --- | --- | --- | --- | --- | --- | --- | --- | --- | --- | --- | --- | --- | --- | --- | --- | --- | --- | --- | --- | --- | --- | --- | --- | --- | --- | --- | --- | --- | --- | --- | --- | --- | --- | --- | --- | --- |

a. In accordance with CLSI guidelines, ciprofloxacin (Cp) MICs for *Aeromonas* are interpreted as follows: susceptible is ≤1 μg/mL; resistant is ≥ 4 μg/mL
