# Supplementary material for: Low-Level Antimicrobials in the Medicinal Leech Select for Resistant Pathogens That Spread to Patients
Source: mBio. 2018 Jul 24;9(4):e01328-18. doi: 10.1128/mBio.01328-18 (PMC6058295; doi:10.1128/mBio.01328-18)
Supplement: TABLE S8 [file mbo004183985st8.docx]

**Supplementary Table 8. Primer Sequences for Illumina *gyrA* deep sequencing**

| Forward Primers | | Reverse Primers | |
| --- | --- | --- | --- |
|  | |  | |
| Seq Name | Sequence 5'->3' | Seq Name | Sequence 5'->3' |
| gyrA_F_ilm_R21x1 | CAAGCAGAAGACGGCATACGAGAT**CGTGAT**GTGACTGGAGTTCAGACGTGTGCTCTTCCGATCTTGGGGGAACGACTGGAACAAG | gyrA-547R_A | AATGATACGGCGACCACCGAGATCTACACTCTTTCCCTACACGACGCTCTTCCGATCT**ACGTCATG**TAGRATGTTGGTCGCCATRCC |
| gyrA_F_Ilm_R21x3 | CAAGCAGAAGACGGCATACGAGAT**GCCTAA**GTGACTGGAGTTCAGACGTGTGCTCTTCCGATCTTGGGGAACGACTGGAACAAG | gyrA-547R_B | AATGATACGGCGACCACCGAGATCTACACTCTTTCCCTACACGACGCTCTTCCGATCT**CATGACGT**TAGRATGTTGGTCGCCATRCC |
| gyrA_F_Ilm_R21x4 | CAAGCAGAAGACGGCATACGAGAT**TGGTCA**GTGACTGGAGTTCAGACGTGTGCTCTTCCGATCTTGGGGAACGACTGGAACAAG | gyrA-547R_C | AATGATACGGCGACCACCGAGATCTACACTCTTTCCCTACACGACGCTCTTCCGATCT**GCATCGTA**TAGRATGTTGGTCGCCATRCC |
| gyrA_F_Ilm_R21x5 | CAAGCAGAAGACGGCATACGAGAT**CACTGT**GTGACTGGAGTTCAGACGTGTGCTCTTCCGATCTTGGGGAACGACTGGAACAAG | gyrA-547R_D | AATGATACGGCGACCACCGAGATCTACACTCTTTCCCTACACGACGCTCTTCCGATCT**TACGATGC**TAGRATGTTGGTCGCCATRCC |
| gyrA_F_Ilm_R21x6 | CAAGCAGAAGACGGCATACGAGAT**ATTGGC**GTGACTGGAGTTCAGACGTGTGCTCTTCCGATCTTGGGGAACGACTGGAACAAG | gyrA-547R_E | AATGATACGGCGACCACCGAGATCTACACTCTTTCCCTACACGACGCTCTTCCGATCT**ATGCTACG**TAGRATGTTGGTCGCCATRCC |
| gyrA_F_Ilm_R21x7 | CAAGCAGAAGACGGCATACGAGAT**GATCTG**GTGACTGGAGTTCAGACGTGTGCTCTTCCGATCTTGGGGAACGACTGGAACAAG | gyrA-547R_F | AATGATACGGCGACCACCGAGATCTACACTCTTTCCCTACACGACGCTCTTCCGATCT**CGTAGCAT**TAGRATGTTGGTCGCCATRCC |
|  |  |  |  |
| gyrA_F_Ilm_R21x8 | CAAGCAGAAGACGGCATACGAGAT**TCAAGT**GTGACTGGAGTTCAGACGTGTGCTCTTCCGATCTTGGGGAACGACTGGAACAAG | gyrA-547R_G | AATGATACGGCGACCACCGAGATCTACACTCTTTCCCTACACGACGCTCTTCCGATCT**GTACTGCA**TAGRATGTTGGTCGCCATRCC |
| gyrA_F_Ilm_R21x9 | CAAGCAGAAGACGGCATACGAGAT**CTGATC**GTGACTGGAGTTCAGACGTGTGCTCTTCCGATCTTGGGGAACGACTGGAACAAG | gyrA-547R_H | AATGATACGGCGACCACCGAGATCTACACTCTTTCCCTACACGACGCTCTTCCGATCT**TGCAGTAC**TAGRATGTTGGTCGCCATRCC |
| gyrA_F_Ilm_R21x10 | CAAGCAGAAGACGGCATACGAGAT**AAGCTA**GTGACTGGAGTTCAGACGTGTGCTCTTCCGATCTTGGGGAACGACTGGAACAAG | gyrA_R_Ilm_R21x12 | AATGATACGGCGACCACCGAGATCTACACTCTTTCCCTACACGACGCTCTTCCGATCTNNNNGRATGTTGGTCGCCATRCC |
| gyrA_F_Ilm_R21x11 | CAAGCAGAAGACGGCATACGAGAT**GTAGCC**GTGACTGGAGTTCAGACGTGTGCTCTTCCGATCTTGGGGAACGACTGGAACAAG |  |  |
| gyrA_F_Ilm_R21x12 | CAAGCAGAAGACGGCATACGAGAT**CGTGAT**GTGACTGGAGTTCAGACGTGTGCTCTTCCGATCTTGGGGAACGACTGGAACAAG |  |  |
